# Identification of 42 Genes Linked to Stage II Colorectal Cancer Metastatic Relapse

**DOI:** 10.3390/ijms17050598

**Published:** 2016-04-28

**Authors:** Rabeah A. Al-Temaimi, Tuan Zea Tan, Makia J. Marafie, Jean Paul Thiery, Philip Quirke, Fahd Al-Mulla

**Affiliations:** 1 Human Genetics Unit, Department of Pathology, Faculty of Medicine, Kuwait University, Kuwait 13110, Kuwait; rabeah@hsc.edu.kw; 2 Cancer Science Institute of Singapore, National University of Singapore, Singapore 117599, Singapore; csittz@nus.edu.sg; 3 Kuwait Medical Genetics Center, Maternity Hospital, Kuwait 13001, Kuwait; mj_marafie@yahoo.com; 4 Department of Biochemistry, National University of Singapore, Singapore 117599, Singapore; bchtjp@nus.edu.sg; 5 Pathology and Tumour Biology, Leeds Institute of Cancer and Pathology, University of Leeds, Leeds LS9 7TF, UK; p.quirke@leeds.ac.uk; 6 Molecular Pathology Unit, Department of Pathology, Faculty of Medicine, Kuwait University, Kuwait 13110, Kuwait; fahd@al-mulla.org

**Keywords:** colorectal cancer, microarray, stage II, copy number aberrations, disease free survival, gene expression, metastasis

## Abstract

Colorectal cancer (CRC) is one of the leading causes of cancer mortality. Metastasis remains the primary cause of CRC death. Predicting the possibility of metastatic relapse in early-stage CRC is of paramount importance to target therapy for patients who really need it and spare those with low-potential of metastasis. Ninety-six stage II CRC cases were stratified using high-resolution array comparative genomic hybridization (aCGH) data based on a predictive survival algorithm and supervised clustering. All genes included within the resultant copy number aberrations were each interrogated independently at mRNA level using CRC expression datasets available from public repositories, which included 1820 colon cancers, and 167 normal colon tissues. Reduced mRNA expression driven by copy number losses and increased expression driven by copy number gains revealed 42 altered transcripts (29 reduced and 13 increased transcripts) associated with metastatic relapse, short disease-free or overall survival, and/or epithelial to mesenchymal transition (EMT). Resultant genes were classified based on gene ontology (GO), which identified four functional enrichment groups involved in growth regulation, genomic integrity, metabolism, and signal transduction pathways. The identified 42 genes may be useful for predicting metastatic relapse in stage II CRC. Further studies are necessary to validate these findings.

## 1. Introduction

Microarray comparative genomic hybridization (aCGH) has been extensively used to profile colorectal cancer (CRC) for copy number aberrations. However, their direct relevance to prognosis and therapeutic interventions has remained elusive [1,2]. Undoubtedly, early cancer detection contributes to a better patient outcome. Fecal occult blood testing, fecal immunochemical/molecular testing, and colonoscopy-based screening have become wide-spread in developed countries as a preventive measure for the detection of CRC in its early stages. However, 10%–30% of early-stage CRC still relapse with metastases within two years of surgical treatment [3,4]. To address this critical issue, various technologies such as aCGH, metaphase, and next-generation sequencing were employed in attempts targeted towards the identification of suitable prognostic biomarkers in early stage CRC. Array-based analysis of DNA copy number aberrations (CNAs), in particular, has proven to be very effective in identifying recurrent CNAs in CRC. It is usually argued that CNAs of recurrent nature may imply their evolutionary importance in driving CRC progression [5,6]. For this reason, large bodies of publications have attempted to ascertain early stage CRC patients at risk of metastatic relapse based on the genomic profiling of primary tumors. However, such attempts rarely identified reliable and replicable CNAs or genes predictive of survival. Several predictive gene signatures have been reported for CRC [7,8]. Yet, these studies suffer limitations such as small sample size and lack of adequate expression-based validation. This is not surprising given that most CNAs encompass large genomic aberrations that usually harbor hundreds of bystander genes among which only few genes may be potentially useful for prognostication [8]. Therefore, validation studies linking genomic aberrations at the DNA level to transcription have been limited [7]. The two questions that need to be addressed are; first, how can we identify key “driver” CNA events from the enormous amount of random “passenger” CNAs that have no functional significance? Second, how can we pinpoint the few metastasis suppressor genes residing within large areas of genomic deletions? Here, we attempted to address these questions. Firstly, we identified CNAs exclusively found in stage II metastatic CRC using stringent experimental and statistical algorithms. We then grouped the CNAs generated based on their significant effects on disease survival. The resultant list of potential metastasis-associated genes (958 genes) residing within CNA regions were each independently validated for aberrant expression in publicly available CRC cohorts’ expression data. This validation identified 42 genes, potentially involved in metastasis. Twenty-nine metastasis suppressor genes whose copy number deletions resulted in diminished transcription, and reduced overall survival (OS) or disease-free survival (DFS), and 13 potentially metastasis-enhancing genes whose copy number gains and overexpression associated with metastatic relapse, short disease-free or overall survival or/and epithelial to mesenchymal transition (EMT). A large and randomized study focused on the 42 genes to confirm our data and validate their clinical utility is now warranted.

## 2. Results

### 2.1. CRC aCGH Profile

Table 1 illustrates the clinicopathological characteristics of 96 stage II CRC cases investigated in our study. The data show that pathological classification alone has limited efficacy in distinguishing subclasses of stage II CRC in terms of aggressiveness or prognosis.

**Figure 1:**
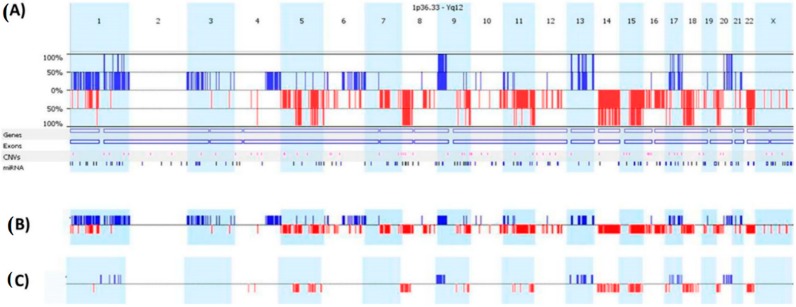
Copy number aberrations in CRC stage II using the first analytical approach STAC (**A**), which compares the chromosomal copy numbers found in metastatic CRC with non-metastatic CRC; and the second analytical approach; the survival predictive algorithm (**B**), which identifies chromosomal copy number gains associated with reduced disease-free survival; (**C**) shows the frequencies of the aberrations in the two approaches and identifies CNA common to both methods (100%). Each column represents a chromosome with blue bars indicating copy number gains and red bars copy number losses.

To refine our search for genes involved in metastasis, we excluded seven cases that later presented with local recurrence. Significance testing for aberrant copy number (STAC) analysis focused on chromosomal CNAs of 89 CRC cases, which produced acceptable derivative of log ratio spread (DLRS) values below 0.5. DLRS is defined as the spread of the log ratio differences between consecutive probes along all chromosomes. Comparison using stringent criteria between CNAs found in 11 stage II CRC with metastatic disease and CNAs found in 78 stage II CRC cases that remained disease-free resulted in the identification of chromosomal CNAs significantly enriched in metastatic CRC (Figure 1a). The most frequent copy number gains (CNGs) were in chromosomes seven (56%), 8q (56%), 13q11-q34 (61%), and 20q11.1-q13.33 (79%). Other less frequent regional gains include; chromosomes 1q, 9p, and 17q. The most frequent copy number losses (CNLs) were in chromosome arms 1p (71%), 8p (72%), 17p (55%), 22q (60%), and chromosomes 14 (77%), 15 (66%), and 18 (80%). To narrow down genes with predictive potential, a second analytical approach that is dependent solely on survival data and not whether the case relapsed with metastases or not was utilized to point out CNAs shared by CRC cases with reduced DFS (Figure 1b). The two approaches were compared to compile a common CNA profile that may influence metastasis and DFS (Figure 1c). The most significant chromosomal gains that are consistently associated with metastatic CRC recurrence and poor survival were gains in chromosomes 1q, 9p, 13q11-q34, 17q, and 20q11.1-q13.33; and losses in chromosomes 5q12.1-q35.3, 8p12-p23.3, 9q33.1-q34.3, 11q23.3-q25, 14q11.2-q32.31, 15q21.1-q26.3, 18p11.31-q21.1, 20p12.1-p13, and 22q11.21-q13.31. There were 1099 genes located within significant CNAs that were further scrutinized for their existence in normal healthy individuals [9,10]. CNAs frequent in healthy normal genomes that overlapped 5%–100% with mapped CRC metastatic *vs.* non-metastatic chromosomal gains were excluded from further analysis. Interestingly, all CNAs, except for 8p chromosomal region harboring SPAG11A, had minimal overlap with common CNAs in normal healthy individuals. This elimination step resulted in 958 candidate genes for further investigation (Table S1). While some of the CNAs housed one to a few genes, larger CNAs contained many genes that may modify cancer progression or are more likely “innocent passenger” CNAs that are of no functional significance. In addition, CNA profiles segregated according to our cohort’s clinicopathological characteristics also resulted in significant clustering into identifiable groups. Distinct CNAs were found between microsatellite stable (MSS) and microsatellite instable (MSI) CRC, and other clinicopathological characteristics (manuscripts submitted elsewhere). However, we chose to focus here on metastasis associated CNAs. Consequently, the expression level of each of the 958 genes resultant from metastatic CRC CNAs profile was interrogated independently using publicly available CRC datasets to validate their clinical relevance based on; their differential expression in normal and CRC samples, association with grade, microsatellite instability, stage, DFS, overall survival (OS), and EMT score.

### 2.2. Expression Analysis of Candidate Oncogenes

Approximately 307 genes were not represented on the Affymetrix U133Plus2 platform (Santa Clara, CA, USA), of which 166 genes had probes on U133A platform. Genes without a probe on either platform were mostly miRNA genes and were excluded. At first, two categorical variables were considered, DFS and OS to select CNAs’ genes of altered expression. This was done to isolate genes of strong prognostic value. Interrogating gene expression changes driven by CNAs revealed 42 altered transcripts (29 reduced and 13 increased) associated with metastatic relapse, short DFS, or OS (Table 2).

**Figure 2:**
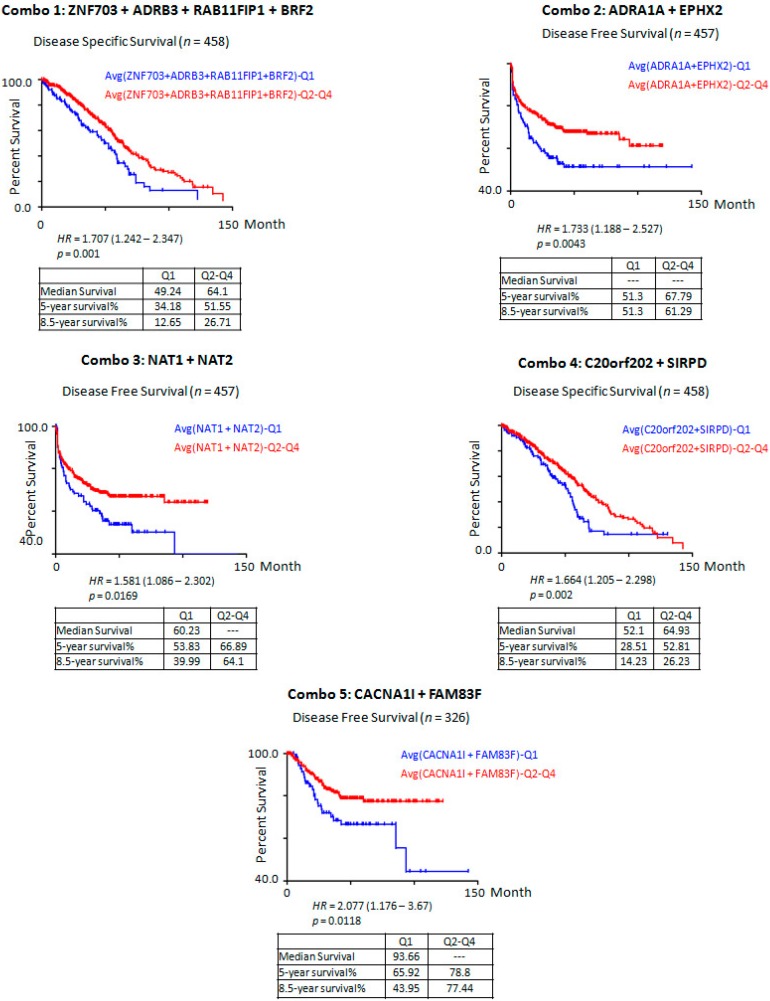
Five different combinations (Combo 1-5) of candidate tumor suppressor genes consistently consolidated their association with disease specific survival (OS), and disease free survival (DFS) in CRC samples’ expression data. Q1–Q4 signify expression quartiles with Q1 being the lowest and Q4 the highest expression quartile.

Our findings show that several candidate oncogenes of significant prognostic potential reside within regions of chromosomal gains and are overexpressed, most likely, under the influence of gene amplification within these regions. Moreover, our results pinpointed metastasis-suppressor genes of significant prognostic potential residing within chromosomal loss areas, and are underexpressed due to genetic deletions. To further refine our list for genes with the greatest prognostic power, we selected genes significantly associated with DFS or OS using a stringent criterion (*p*-value < 0.005), and compared their expression levels in tumor against normal colon tissues. A total of 19 genes had highly significant associations with DFS or OS, of which only 14 had significantly differential expression in normal colon *vs.* CRC tissues (Figure S1). Therefore, these genes have the greatest discriminatory application in clinical prognostication of stage II CRCs at risk of metastatic relapse. We next focused on evaluating the combined potential of a set of deleted genes in relation to their combined effects on DFS or OS. Our hypothesis was that double or more deletions of candidate tumor suppressor genes will significantly increase their effect on DFS and OS in CRC patients. Combination analysis results show that while single deleted gene reduced expression associated with a significant reduction in DFS or OS, their combined underexpression analysis in CRC strengthened their prognostic potential in association with reduced DFS/OS (Figure 2).

Lastly, we analyzed each of the 42 genes’ expression levels in relation to the clinicopathological characteristics of analyzed CRC samples from the GEO database, which included; tumor grade, cancer stage (stages I–IV), expression in MSS *vs.* MSI tumors, expression levels in tumors *vs.* normal colon tissues, and their relationship to EMT in cancers. GEO database clinicopathological correlation data for all of the 29 potentially metastasis-suppressor genes and the 13 potentially metastasis-enhancer genes are shown in (Tables S2–S9).

## 3. Discussion

CRC represents a heterogeneous group of gastrointestinal neoplasia. Stage II CRC is the most clinically challenging stage as it is the critical point between remission and progression into aggressive stages. Stage II CRC has 20%–30% risk of metastasis, whereas stage III CRC has a 50%–80% risk of distant metastasis [11]. The genetic complexity of stage II CRC is reflected by its extensive CNA profile that presents an additional limitation of sifting the “culprit” genes from the “innocent passenger” ones. The ongoing theory is that cancer metastases arise due to the acquisition of the metastatic phenotype, which appears to have an underlying metastatic genotype [12,13]. We generated genomic CNA profiles for stage II CRCs, with and without subsequent metastatic disease to identify CNAs associated with metastasis or reduced OS/DFS in stage II CRC. We then utilized publicly-available high-density oligonucleotide-based microarray expression data to study the effect of these CNAs. The use of well-defined expression profiles from sets of colorectal cancers may serve as reliable and focused validation cohorts. Our resultant genes were individually scrutinized for association with cancer biology. In addition, a stringent gene selection method was employed to exclude false positive CNAs that occur in normal colon samples, and exclude genes whose expression levels in CRC were comparable to normal colon tissues. All 42 genes’ expression had significant association in predicting OS and/or DFS (Table 2). Functional annotation of each gene confirms that each of these genes partake in key processes of cancer progression and metastasis, providing insights into the genetic mechanisms driving metastatic CRC. The 42 genes were divided into genes deleted (29 genes) and genes amplified (13 genes) of which 26 deleted genes and 13 amplified genes had specific known functions. Each gene was literature-researched for reported evidence of known biological function(s) and involvement in cancer.

In the deleted genes list several genes had known tumor suppressor and anti-metastatic properties (Table S10). *CSMD1*, *FAM83F*, and *EPHX2* are known to be silenced, mutated, or deleted in gastrointestinal cancers though their precise functions remain unknown [14,15]. ADRA1A, APOBEC3D, CABIN1, DIO3, GP1BB, HNF6, MCM8, PCDHGA11, WDR5, ZNRF3, and ZNF366 all have functional attributes that are known to control and affect the transition into metastatic pathways. However, *CABIN1* did not significantly associate with our EMT potential analysis (*p* = 0.8), and associated with distinguishing MSS CRC (*p* < 0.05, Tables S4 and S5). *CABIN1* is a pro-apoptotic gene, which suggests that its reduced expression is an early marker for CRC’s proliferation and genomic instability [16]. EMT fundamentally involves genomic and phenotypic changes of epithelial cells into mesenchymal cells, loss of epithelial cell-cell contact, loss of cell polarity, and acquisition of migratory characteristics facilitating metastasis [17]. *ADRA1A* and *ZNF366* have proliferation regulatory functions that limit tumor cells from forming tumors/metastases in response to stimulators [18,19]. Genomic instability and loss of epithelial genomic signatures are also a requirement for EMT. Three deleted signature genes; *APOBEC3D*, *MCM8* and *WDR5* have genomic stability, genomic fidelity, and epigenetic fidelity functions, respectively [20,21,22]. Downregulation of cell-cell contact to facilitate EMT and tumor mass blood transport is achieved by deletion of epithelial cell adhesion regulatory genes that include *GP1BB* and *PCDHGA11* in our resultant deleted genes list [23,24]. *DIO3* is a metabolic suppressor, which has cancer growth rate-limiting properties and may mitigate EMT [25]. Lastly, *ZNRF3* and *HNF6* are EMT and cell-differentiation regulators under normal conditions and are tumor suppressor genes inhibiting the EMT pathway [26,27]. Four deleted genes had contradictory functions to EMT inhibition but had a negative EMT score based on our analysis. These genes associated with other characteristics of CRC based on their expression analysis. *ADRA1D* was differentially expressed in stage IV compared to stages II and III. *NAT1* and *NAT2*, drug metabolism genes; whose expression was associated with stage I MSS CRC. *BRF2*, a pro-proliferation gene, had a positive EMT score and its expression was associated with MSS CRC. Activation of EMT was shown to reduce cell proliferation by targeting specific pro-proliferation genes [28,29]. It is possible that the loss of BRF2 functions in the primary tumor would result in the activation of alternative proliferation pathways in distant metastases during reversion of EMT. The remaining nine deleted genes were of unknown functions, albeit associating with several characteristics of CRC (Tables S2–S5), which warrants further functional studies of these genes.

All amplified genes had known functions, except for *ANXA2P2*, which is a pseudogene for the mesenchymal marker Annexin A2. Most amplified genes had positive association with EMT except for *SEMG1*, *SMU1* and *ING1*. *SMU1* and *ING1* regulate genomic integrity and cell growth and their function could be altered by aberrant expression or gene structural changes that resulted in their association with MSS CRC [30,31]. Similarly, *SEMG1* expression, a negative regulator of calcium import, is associated with MSS CRC. Metabolic advantage is a feature of metastatic tumors and might be mediated by overexpression of *VLDLR*, *USP32*, and *PITPINC1* [32,33,34]. Although *PITPINC1* had a non-significant EMT score, its expression was associated with MSS CRC. Five amplified genes were involved in multiple signaling cascades that can have pleiotropic effects. Those genes were *DOK5*, *DUSP14*, *GLIS3*, *PITPNC1*, and *SEMG1*. These genes are partly characterized for their involvement in cancer and would require further studies. Two amplified genes had contradictory functions to being overexpressed in metastatic cancer, *SCEL* (a keratinocyte differentiation factor), and *MPDZ* (a cell adhesion factor). However, both genes are involved in recruitment and assembly of proteins to cellular surfaces promoting protein-protein interactions. Aberrant expression of these proteins may be involved in the formation of invadopodia, which facilitates invasion of the local extracellular matrix and basement membranes [35,36]. Lastly, it should be noted that our study here is limited to estimating copy number aberrations; it does not discern sequence mutations or epigenetic changes.

**Figure 3:**
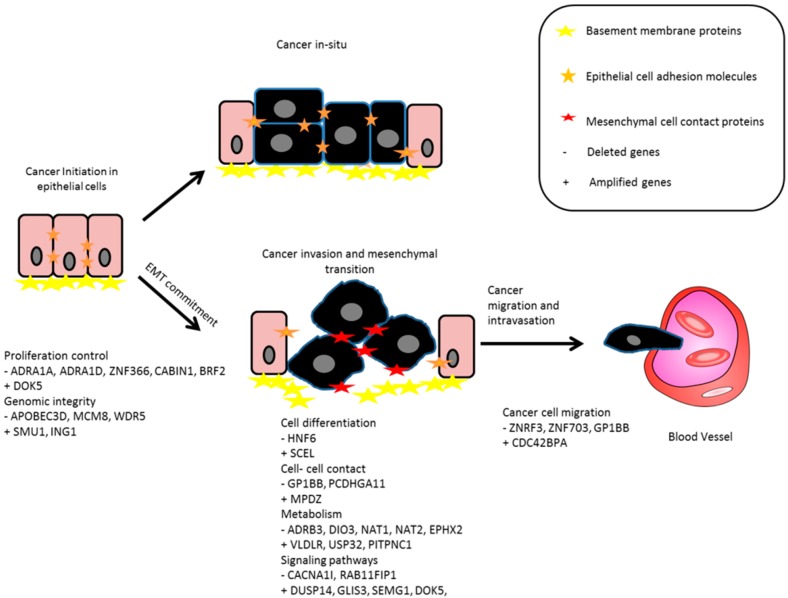
Proposed simple diagram depicting the functional involvement of signature genes in cancer progression, EMT, invasion, and metastasis. The arrows indicate the transition and progression of tumor cells from one stage into the next.

In conclusion, our study characterized the personal nature of stage II CRC CNAs highlighting key genes involved in promoting CRC metastasis. Resultant genes were mostly involved in cell growth regulation, metabolic regulation, and signaling pathways (Figure 3). We have identified some novel candidate genes of possible prognostic value for both overall survival and DFS. As well as novel genes that can be used for CRC classification. These genes can offer new targets for diagnostic and therapeutic designs. We anticipate validating our findings in separate retrospective and prospective studies for their efficiency in predicting metastatic stage II CRC.

## 4. Materials and Methods

### 4.1. CRC Samples

DNA extracted from formalin-fixed paraffin-embedded (FFPE) tissues from 96 patients with sporadic early stage II CRC were used for genomic profiling. Genomic DNA was isolated from microdissected FFPE CRC tissues as described previously [37].

### 4.2. Microsatellite Instability Analysis

Microsatellite fragment analysis was performed on FFPE-extracted DNA using MSI Analysis System Version 1.2 kit (Promega, Madison, WI, USA). Spectral calibration was carried out on the Applied Biosystems 3130 genetic analyzer using the Powerplex Matrix Standards 3100/3130 kit (Promega). Promega’s MSI Analysis System includes fluorescently labeled primers for amplification of seven markers; five mononucleotide repeat markers (Bat-25, BAT-26, NR-21, NR-24, and MONO-27), and two pentanucleotide repeat markers (Penta C and D). Mononucleotide markers are used to determine the MSI status, whereas pentanucleotide markers are used to detect potential sample mix-up by validating that tumor and matching normal samples are from the same individual. DNA concentrations of 10–20 ng from normal and tumor samples were used for the fluorescent PCR-based assay. Microsatellite instability was determined by comparing allelic profiles of microsatellite markers generated by amplification of normal and tumor DNA. Internal lane size standard ILS600 was added to amplified samples to ensure accurate sizing of alleles. A loading cocktail was prepared by mixing the ILS600-PCR product with highly-deionized formamide, and denatured prior to loading onto the 3130 Genetic Analyzer for capillary electrophoresis. The sample’s fragment separation output data were analyzed using GeneMapper software version 4.0 (Applied Biosystems, Foster City, CA, USA). CRC samples were classified as MSS if no marker showed any length variation compared with its matching normal colon mucosa. When two or more of the markers showed length mutation in CRC compared with its matching normal mucosa, the CRC sample was labeled as MSI-high.

### 4.3. Genomic Landscaping Using aCGH

Array CGH was carried out on 96 CRC samples following our standard published protocol [38]. In summary, 2 µg of tumor DNA and sex-matched pooled reference DNA (Promega, Madison, WI, USA) were sonicated in a water bath. The universal linkage system (ULS) Cy3 and Cy5 (Agilent Technologies, Santa Clara, CA, USA) dyes were used to label DNA according to the manufacturer’s protocol. Differentially-labeled DNA was purified by Agilent KREApure columns (Agilent Technologies, Santa Clara, CA, USA). Purified labeled tumor and reference samples were hybridized onto Human Genome CGH arrays 244A slides (Agilent Technologies, Santa Clara, CA, USA) in SureHyb chambers (Agilent Technologies, Santa Clara, CA, USA) for 40 h at 60 °C. Washing and scanning were carried out according to manufacturer’s protocol. Slides were scanned immediately on an Agilent microarray scanner at 5 µm resolution to minimize the impact of environmental factors on signal intensities. Data was extracted from microarray image files using Feature Extraction software (Version 9.5, Agilent Technologies, Santa Clara, CA, USA) and analyzed using Nexus Copy Number™ software (BioDiscovery, El Segundo, CA, USA). Quality values generated from data analysis ranged between 0.05–0.4, and to minimize false positive calls and random copy number variations BioDiscovery’s Fast Adaptive State Segmentation Technique (FASST2) algorithm with a stringent significance threshold of 5.0 × 10^−6^ was used to determine copy number states.

### 4.4. Data Clustering and Statistical Analysis

Traditional means of classifying the importance of cancer-related copy number gain or loss include the frequency of their occurrence in different patients. However, cancer genomes are highly complex and frequently harbor random “passenger” copy number aberrations of no functional significance. To minimize interference from these passenger events, a systematic statistical approach termed significance testing for aberrant copy number (STAC) was employed [39]. The STAC-based algorithm is a robust method, which identifies a set of aberrations that are stacked on top of each other from different samples such that it would not occur by chance. To find these events, aberrations (usually narrow regions) were permutated in each arm of each chromosome and the likelihood for an event occurring at any location at a particular frequency was calibrated. Here, we used STAC to compare CNAs from 11 stage II primary CRC cases with confirmed distant metastases based on a 10-year follow up, and 78 stage II primary CRC relapse-free cases (Table 1). We defined metastatic CRC as distant cancer recurrence away from the primary site. Disease/relapse-free status is defined as no distant metastases (liver, brain, or bone) or local recurrence. We set the likelihood frequency stringently at 35% and a significance *p*-value of <0.05 [39]. To further refine the CNA to include clinically-relevant aberrations, we reanalyzed the data using a survival predictive power statistical approach (SPPS). SPPS correlates CNA with survival time. In this analysis samples with similar CNA regions are grouped (In-Group) and their mean survival calculated and compared to samples without the named CNA (Out-Group). The mean survival times were then compared using the log rank test. This approach highlights CNAs that influence survival time and may be more clinically relevant. Such permutations are not considered in the STAC analysis we used; therefore, the two methods may be considered complementary. Results from both approaches were compared to narrow down the chromosomal regions and resident genes associated with CRC metastasis and disease-free status.

### 4.5. Data Preprocessing of Affymetrix Microarray Gene Expression

Gene expression microarray data of colon cancer on U133A or U133Plus2 platform (Affymetrix, Santa Clara, CA, USA) were downloaded from Gene Omnibus (GEO), including synchronous and metachronous liver metastases from CRC (GSE10961, *n* = 18), primary colorectal tumors (GSE13067, *n* = 74), primary CRCs (GSE13294, *n* = 155), primary CRCs (GSE14333, *n* = 290), colon adenomas and CRCs (GSE15960, *n* = 12, normal = 6), CRCs (GSE17536, *n* = 177 of which 144 are stage II and III), metastatic CRCs (GSE17537, *n* = 55), stage II CRCs (GSE18088, *n* = 53), stage II and III CRCs (GSE18105, *n* = 77, normal = 34), colon adenomas and CRCs (GSE20916, *n* = 101, normal = 44), CRCs (GSE23878, *n* = 35, normal = 24), MSI CRCs (GSE24514, *n* = 34, normal = 15), MSI CRCs (GSE26682, *n* = 331), stage II and III CRCs (GSE31595, *n* = 37), primary stage II CRCs (GSE33113, *n* = 90), primary CRC tumors (GSE35896, *n* = 62), serrated and conventional colorectal adenocarcinoma tumors (GSE4045, *n* = 37), metastatic CRCs (GSE5851, *n* = 80), colorectal adenomas (GSE8671, *n* = 32, normal = 32), and early stage CRC tumors (GSE9348, *n* = 70, normal = 12). Robust Multichip Average normalization was performed on each dataset using R version 2.15.3, Bioconductor Affy package version 1.38.1 (Affymetrix, Santa Clara, CA, USA). The normalized data was compiled and subsequently standardized using ComBat to remove batch effects [40]. The standardized data yielded a meta-cohort of 1820 colon carcinoma, and 167 normal colon tissues. Note that some of the genes are only available on Affymetrix U133Plus2 platform (*n* = 1436), a subset of the meta-cohort. To focus on the effect of tumor suppressor genes, we co-analyzed different combinations of copy number deleted genes in relation to their effect on OS and DFS. Deleted gene combinations depended on their co-presence of their probes in any of the Affymetrix platforms used, and their co-underexpression in a given CRC sample. We stratified expression levels into quartiles (Q) where the first quartile (Q1) is the expression level at the 25th percentile; second quartile is the median or the 50th percentile; the third quartile is the 75th percentile; and, the last quartile (Q4) is the expression levels of the highest 25 percentile.

### 4.6. Estimation of Epithelial-Mesenchymal Transition Score

Derivation of EMT signature and estimation of EMT score were described previously [41]. Briefly, a curated EMT gene set [42], after removing basal keratins genes [43,44], was used as an initial selection of epithelial and mesenchymal cell lines. The first step was to establish an EMT signature using binary regression (BinReg) 2.0 [45] comparing profiles of colon cell lines with low or high EMT enrichment score. Briefly, BinReg uses a Bayesian statistical analysis to fit a binary probit regression model on training data given a set of genes that are most correlated with the EMT phenotype. The regression coefficients of these genes indicate the discriminating power of the gene and are weights for the overall meta-gene profile. The overall meta-gene profile is subsequently used for comparison and predicts the status of the phenotype of the new sample or dataset. In the second step, the BinReg colon cancer EMT signature was applied to predict the EMT status of colon cancer tumor or cell lines. In the third step the extreme 25% samples with the highest probabilities for epithelial or mesenchymal phenotype were used to obtain the epithelial or mesenchymal specific gene list for the colon cancer tumors or cell lines using Significance Analysis of Microarray (SAM) *q*-value = 0 and receiver-operating characteristics curve (ROC) value of 0.85. SAM computed the expression fold change of the genes based on the EMT phenotype, and assessed the significance by comparing the observed fold change against the background expression fold change estimated from 1000 permutations of the samples’ EMT phenotypes. On the other hand, ROC method constructs a ROC curve assessing the predictive power of a gene with respect to the EMT phenotypes. In the fourth step, single-sample GSEA (ssGSEA) was employed to compute the enrichment score of a tumor or cell line based on the expression of the colon cancer tumor- or cell line-specific epithelial or mesenchymal signature genes [46]. In ssGSEA, a Kolmogorov-Smirnov-based method, the enrichment of a signature is estimated by comparing the empirical cumulative distribution function of genes defined by the signature, *vs.* the genes not in the signature. EMT score is defined as the normalized subtraction of the mesenchymal from epithelial enrichment scores. The EMT score is an estimate for the cell line status as epithelial or mesenchymal phenotype with −1.0 indicates fully epithelial and +1.0 fully mesenchymal.

### 4.7. Statistical Analysis

Statistical significance evaluation by Mann-Whitney and Spearman correlation tests were computed using Matlab^®^ R2012a (MathWorks, Natick, MA, USA). Dot plot and Kaplan-Meier analysis were done using GraphPad Prism version 5.04 (GraphPad, La Jolla, CA, USA) or SPSS V6.5 (IBM, New York City, NY, USA).

## Figures and Tables

**Table 1:** Clinicopathological characteristics of stage II CRC cohort.

Patients’ Characteristics		Stage II Who Stayed Disease Free	Stage II with Local Recurrences	Stage II Who Relapsed with Distant Metastasis	*p* Value ^a^
Mean age in years		64.4	75.5	75.4	0.004 ^b^
Sex	Male	41	2	5	0.43
	Female	37	5	6	
Total		78	7	11	
Site	Right	19	2	1	0.16
	Left	28	1	9	
	Rectum	16	1	1	
	Unknown	15	3	0	
T-stage	T3	44	3	9	0.27
	T4	19	4	2	
	Unknown	15	0	0	
Differentiation	Well	10	1	1	0.8
	Moderate	56	5	9	
	Poor	5	1	0	
	Unknown	7	0	1	
MMR status	MSI	14	1	0	0.45
	MSS	59	5	9	
	Unknown	5	1	2	
Follow-up	Mean DFS	9.5 years	3.9 years	3.08 years	

^a^ Fisher’s exact test; ^b^ one-way ANOVA. DFS is disease free survival; MSI is microsatellite unstable; MSS is microsatellite stable; MMR is mismatch repair.

**Table 2:** Cox regression for survival analysis of the 42 genes’ mRNA expression in meta-cohort (Affymetrix U133A, *n* = 1820; or U133Plus2, *n* = 1436) for overall survival (OS), and disease-free survival (DFS). A negative regression coefficient implies a better prognosis for patients if they retain the function of a given gene (worse prognosis when the cancer underexpresses the gene). Conversely, a positive regression coefficient means that the hazard is higher for a given gene’s overexpression and, thus, the prognosis worse.

Gene	Gene ID	aCGH CNA Event	%CNA Overlap with Normal	Cox’s OS (*p*-Value)	Cox’s DFS (*p*-Value)
*ADRA1A*	148	Loss	0.42	−0.40185, (0.152322)	−1.67582, (0.001948)
*ADRA1D*	146	Loss	1.15	−0.5968, (0.020507)	0.066504, (0.880385)
*ADRB3*	155	Loss	0	−1.21809, (0.002366)	−1.12818, (0.08601)
*APOBEC3D*	140564	Loss	0	−0.45803, (0.24853)	−1.56085, (0.0224)
*BRF2*	55290	Loss	0	−0.45526, (0.02013)	0.061887, (0.8515)
*C20orf202*	400831	Loss	0.89	−0.80141, (0.000481)	−0.19907, (0.628292)
*CABIN1*	23523	Loss	4.02	−0.47782, (0.0210)	−0.51601, (0.14935)
*CACNA1I*	8911	Loss	0	−0.69524, (0.00404)	−0.73386, (0.082677)
*CSMD1*	64478	Loss	0	−0.01578, (0.956357)	−1.30789, (0.01948)
*DIO3*	1735	Loss	1.76	−0.32561, (0.041777)	−0.00829, (0.973719)
*EPHX2*	2053	Loss	0.42	0.036582, (0.61411)	−0.30515, (0.0099)
*FAM83F*	113828	Loss	0	−0.13746, (0.287004)	−0.72925, (0.00085)
*GP1BB*	2812	Loss	0	−0.56827, (0.000246)	−0.19289, (0.495577)
*KIAA1656*	85371	Loss	0	−0.60241, (0.01922)	−1.02118, (0.032326)
*LOC339593*	339593	Loss	0.17	0.29021, (0.385267)	−1.12678, (0.047789)
*MCM8*	84515	Loss	0	0.043419, (0.614947)	−0.31948, (0.034203)
*NAT1*	9	Loss	0	0.028159, (0.713606)	−0.36337, (0.007197)
*NAT2*	10	Loss	0	−0.09059, (0.160865)	−0.30169, (0.003804)
*HNF6*	3175	Loss	0	−0.66924, (0.017955)	−0.51152, (0.31)
*PCDHGA11*	56105	Loss	0	−0.85799, (0.001447)	−0.577, (0.33928)
*RAB11FIP1*	80223	Loss	0	−0.38036, (5.55× 10^−05^)	−0.02196, (0.87)
*SPAG11A*	653423	Loss	68.65	−0.81665, (0.001447)	−0.13298, (0.57)
*SIRPD*	128646	Loss	0.89	−0.89166, (0.014166)	−0.13392, (0.82)
*TEX43*	389320	Loss	0	−0.46611, (0.175471)	−0.19907, (0.01162)
*TOP1P2*	7152	Loss	0	−1.13965, (0.005692)	0.599602, (0.32)
*WDR5*	11091	Loss	0	−0.0007, (0.995962)	−0.52505, (0.024307)
*ZNF366*	167465	Loss	0.07	−0.76274, (0.01779)	−0.93429, (0.09208)
*ZNF703*	80139	Loss	0	−0.32601, (0.000744)	−0.14954, (0.366064)
*ZNRF3*	84133	Loss	0	−0.03445, (0.605797)	−0.33884, (0.002065)
*ANXA2P2*	304	Gain	0	0.478663, (0.003259)	0.997702, (0.000504)
*CDC42BPA*	8476	Gain	0	0.032966, (0.690294)	0.401718, (0.005473)
*DOK5*	55816	Gain	0.57	0.105512, (0.1)	0.577502, (0.002218)
*DUSP14*	11072	Gain	0.70	0.207902, (0.047604)	0.709569, (0.000159)
*GLIS3*	169792	Gain	1.25	0.0916, (0.210004)	0.397791, (0.000566)
*ING1*	3621	Gain	0	0.461934, (0.020587)	0.127394, (0.710857)
*MPDZ*	8777	Gain	0	0.079568, (0.406857)	0.657405, (3.98 × 10^−5^)
*PITPNC1*	26207	Gain	0	0.257758, (0.029048)	0.418362, (0.026674)
*SCEL*	8796	Gain	0	0.081094, (0.202236)	0.301113, (0.000357)
*SEMG1*	6406	Gain	0	0.098826, (0.043099)	0.153704, (0.061048)
*SMU1*	55234	Gain	0.73	0.36857, (0.008896)	0.016381, (0.942047)
*USP32*	84669	Gain	0	0.162429, (0.221638)	0.586012, (0.01236)
*VLDLR*	7436	Gain	0.14	−0.00629, (0.92868)	0.323607, (0.004972)

## Supplementary Materials

Supplementary materials can be found at http://www.mdpi.com/1422-0067/17/5/598/s1.

## Abbreviations

aCGH	Array comparative genomic hybridization
CRC	Colorectal Cancer
CAN	Copy number aberration
OS	Overall survival
DFS	Disease free survival
EMT	Epithelial mesenchymal transition
MSI	Microsatellite instable
MSS	Microsatellite stable
MMR	Mismatch repair
STAC	Significance testing for aberrant copy number
DLRS	Derivative of log ratio spread
CNG	Copy number gain
CNL	Copy number loss
FFPE	Formalin-fixed paraffin embedded
FASST2	Fast Adaptive State Segmentation Technique
SPPS	survival predictive power statistical
BinReg	Binary regression
SAM	Significance analysis of microarray
ROC	Receiver-operating characteristics curve
