# Supplementary material for: Identification of 42 Genes Linked to Stage II Colorectal Cancer Metastatic Relapse
Source: Int J Mol Sci. 2016 Apr 28;17(5):598. doi: 10.3390/ijms17050598 (PMC4881437; doi:10.3390/ijms17050598)
Supplement: Supplementary file 1 [file ijms-17-00598-s001.pdf]

Supplementary Materials: Identification of 42 Genes Linked to Stage II Colorectal Cancer Metastatic Relapse

Rabeah A. Al-Temaimi, Tuan Zea Tan, Makia J. Marafie, Jean Paul Thiery, Philip Quirke and Fahd Al-Mulla

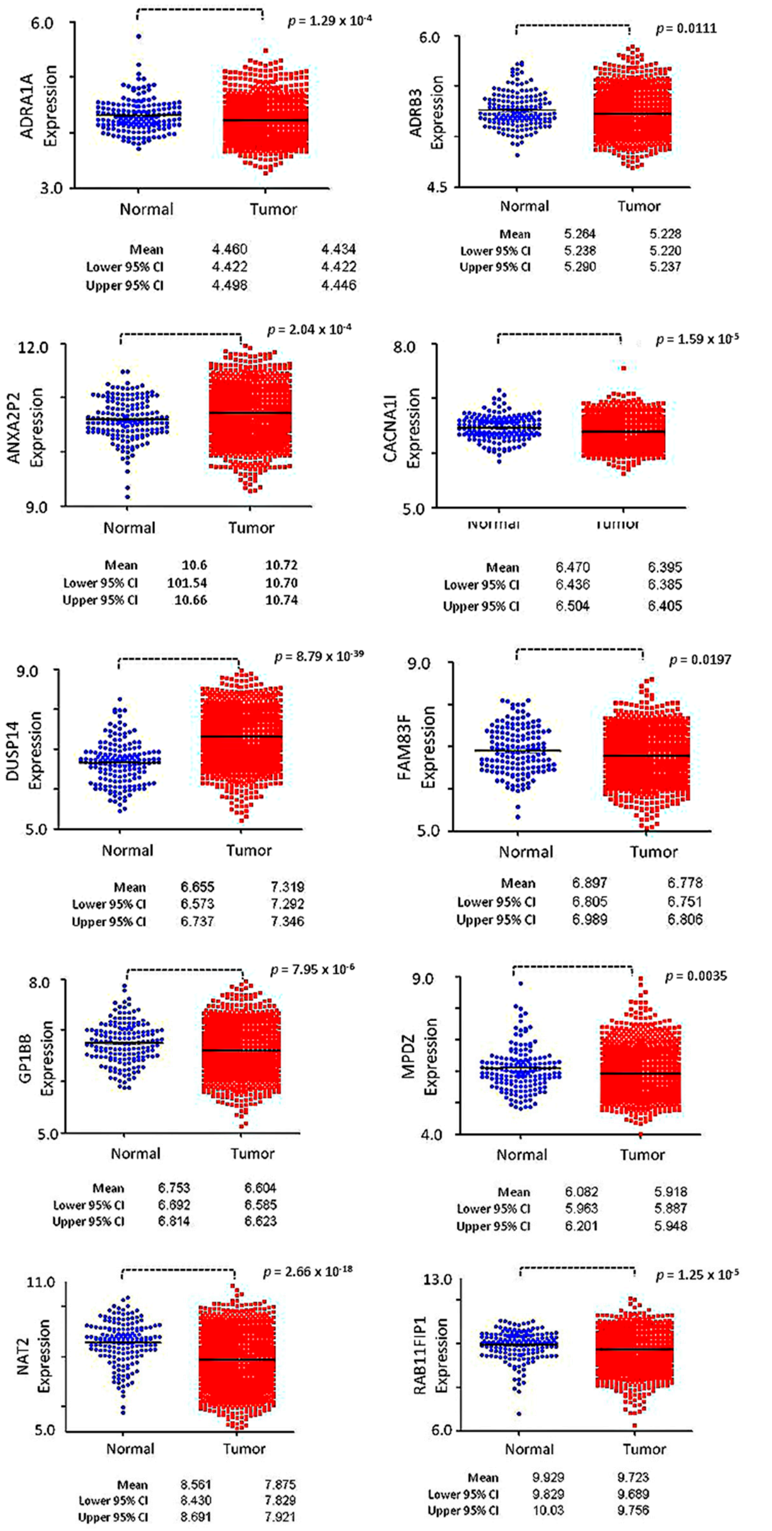

Figure S1. *Cont.*

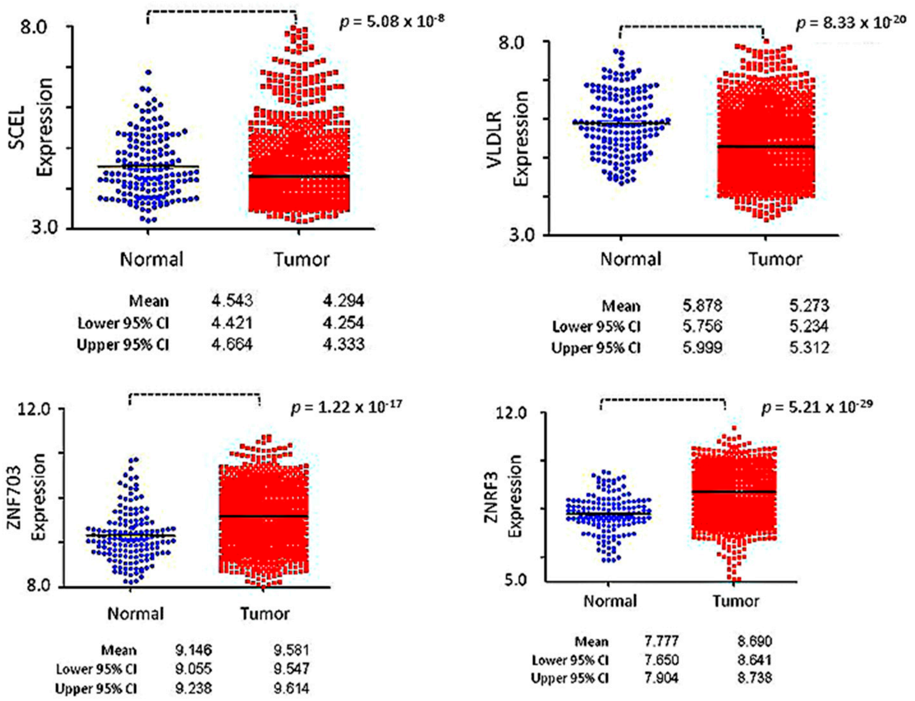

Figure S1. Mean expression levels of fourteen genes of significant association with CRC DFS and OS that are differentially expressed in normal colon compared to CRC tissues. Each dot represents a sample.

Table S1. Copy number aberrations associated with poor disease-free survival and metastasis in early stage II CRC as predicted by STAC and SPPS combined methodologies with resident gene symbols. CN stands for copy number, whereas CNV is copy number variation.

Region	Region Length	Cytoband Location	Event	% of CNV Overlap	Count of Genes	Gene Symbols
**chr1:113,025,076–113,199,133**	174,057	p13.2	CN Loss	0.0	2	*AKR7A2P1, SLC16A1*
**chr1:141,465,960–141,822,265**	356,305	q12–q21.1	CN Gain	95.9	1	*SRGAP2B*
**chr1:144,911,564–146,242,907**	1,331,343	q21.1	CN Gain	99.6	16	*MIR5087, LOC10013000 0, FLJ39739, LOC10028679 3, PPIAL4G, PPIAL4A, NBPF14, NBPF15, NBPF16, PPIAL4E, NBPF16, PPIAL4D, PPIAL4F, LOC645166, LOC388692, FCGR1C*
**chr1:177,209,428–177,226,812**	17,384	q25.3	CN Gain	0.0	0	
**chr1:197,652,888–197,676,831**	23,943	q32.1	CN Gain	0.0	1	*KIF21B*
**chr1:201,015,278–201,033,308**	18,030	q32.1	CN Gain	0.0	1	*PLEKHA6*
**chr1:201,289,154–201,298,247**	9093	q32.1	CN Gain	0.0	0	
**chr1:216,820,186–217,043,421**	223,235	q41	CN Gain	0.0	2	*RAB3GAP2, AURKAPS1*
**chr1:223,586,936–223,618,129**	31,193	q42.13	CN Gain	0.0	1	*CDC42BPA*
**chr1:223,734,287–224,055,935**	321,648	q42.13	CN Gain	0.0	1	*CDC42BPA*
**chr4:93,838,017–93,935,669**	97,652	q22.3	CN Loss	0.0	1	*GRID2*
**chr5:62,861,335–63,175,713**	314,378	q12.1-q12.2	CN Loss	0.0	0	
**chr5:63,276,234–63,281,719**	5485	q12.3	CN Loss	0.0	0	
**chr5:63,824,454–63,890,954**	66,500	q12.3	CN Loss	0.0	1	*RGS7BP*
**chr5:63,982,847–64,092,827**	109,980	q12.3	CN Loss	0.0	2	*FAM159B, SREK1IP1*
**chr5:65,613,783–65,628,119**	14,336	q12.3	CN Loss	0.0	0	
**chr5:66,625,878–66,992,921**	367,043	q12-q13.1	CN Loss	0.1	0	
**chr5:67,038,088–67,070,360**	32,272	q13.1	CN Loss	0.0	0	
**chr5:71,478,793–72,228,866**	750,073	q13.2	CN Loss	0.1	7	*MAP1B, MIR4803, MRPS27, PTCD2, ZNF366, TNPO1, MIR4804*
**chr5:75,418,362–75,787,844**	369,482	q13.3	CN Loss	0.0	2	*SV2C, IQGAP2*
**chr5:76,429,582–76,636,664**	207,082	q13.3	CN Loss	0.0	2	*ZBED3-AS1, PDE8B*
**chr5:76,652,632–76,716,658**	64,026	q13.3	CN Loss	0.0	1	*PDE8B*
**chr5:78,401,367–78,735,677**	334,310	q14.1	CN Loss	0.0	4	*BHMT2, BHMT, JMY, HOMER1*

**chr5:78,900,343–79,945,203**	1,044,860	q14.1	CN Loss	0.1	11	*PAPD4, CMYA5, MTX3, THBS4, SERINC5, LOC644936, SPZ1, CRSP8P, ZFYVE16, FAM151B, ANKRD34B*
**chr5:80,435,819–80,734,178**	298,359	q14.1	CN Loss	0.0	5	*RASGRF2, CKMT2, LOC10013106 7, ZCCHC9, ACOT12*
**chr5:81,139,670–82,114,164**	974,494	q14-q14.2	CN Loss	0.0	3	*ATG10, RPS23,*
						ATP6AP1L
**chr5:82,650,005–83,385,290**	735,285	q14-q14.3	CN Loss	2.5	4	*XRCC4, VCAN, HAPLN1, EDIL3*
**chr5:85,551,789–85,979,590**	427,801	q14.3	CN Loss	0.0	3	*NBPF22P, COX7C, MIR3607*
**chr5:125,906,988–126,007,887**	100,899	q23.2	CN Loss	0.0	3	*ALDH7A1, PHAX, TEX43*
**chr5:126,218,261–126,981,847**	763,586	q23.2	CN Loss	0.0	4	*MARCH3, C5orf63, MEGF10, PRRC1*
**chr5:127,942,415–128,305,543**	363,128	q23.3	CN Loss	0.0	0	
**chr5:132,843,603–133,166,922**	323,319	q31.1	CN Loss	0.0	1	*FSTL4*
**chr5:134,347,761–134,796,875**	449,114	q31.1	CN Loss	0.0	4	*CATSPER3, PITX1, LOC340073, H2AFY*
**chr5:140,069,236–140,199,990**	130,754	q31.3	CN Loss	2.1	10	*VTRNA1–1, VTRNA1–2, VTRNA1–3, PCDHA1, PCDHA2, PCDHA3, PCDHA4, PCDHA5, PCDHA6, PCDHA7*
**chr5:140,219,209–140,485,367**	266,158	q31.3	CN Loss	1.8	19	*PCDHA1, PCDHA2, PCDHA3, PCDHA4, PCDHA5, PCDHA6, PCDHA7, PCDHA8, PCDHA9, PCDHA10, PCDHA11, PCDHA12, PCDHA13,*
						PCDHAC1, PCDHAC2, PCDHB1, PCDHB2, PCDHB3, PCDHB4
**chr5:140,724,660–140,871,691**	147,031	q31.3	CN Loss	0.0	23	*PCDHGA1, PCDHGA2, PCDHGB8P, PCDHGA3, PCDHGB1, PCDHGA4, PCDHGB2, PCDHGA5, PCDHGB3, PCDHGA6, PCDHGA7, PCDHGB4, PCDHGA8, PCDHGB5, PCDHGA9, PCDHGB6, PCDHGA10, PCDHGB7, PCDHGA11, PCDHGA12, PCDHGC3, PCDHGC4, PCDHGC5*
**chr5:143,838,624–144,617,390**	778,766	q31.3-q32	CN Loss	0.0	0	
**chr5:145,868,969–146,052,295**	183,326	q32	CN Loss	1.3	3	*TCERG1, GPR151, PPP2R2B*
**chr5:147,767,845–148,006,323**	238,478	q32	CN Loss	0.0	2	*FBXO38, HTR4*
**chr5:148,050,020–148,670,099**	620,079	q32	CN Loss	0.0	4	*ADRB2, SH3TC2, ABLIM3, AFAP1L1*
**chr5:153,205,713–153,335,688**	129,975	q33.2	CN Loss	0.0	0	
**chr5:156,046,644–156,069,471**	22,827	q33.3	CN Loss	0.0	1	*SGCD*
**chr5:177,692,918–177,924,656**	231,738	q35.3	CN Loss	0.0	1	*COL23A1*
**chr5:178,303,189–178,617,480**	314,291	q35.3	CN Loss	0.7	5	*ZNF454, GRM6, ZNF879, ZNF354C, ADAMTS2*
**chr5:178,726,856–178,908,104**	181,248	q35.3	CN Loss	82.8	0	
**chr5:179,569,883–179,719,152**	149,269	q35.3	CN Loss	0.0	2	*MAPK9, GFPT2*
**chr5:180,560,732–180,581,384**	20,652	q35.3	CN Loss	0.0	1	*TRIM7*
**chr8:0–225,128**	225,128	p23.3	CN Loss	0.0	3	*OR4F21, RPL23AP53, ZNF596*
**chr8:3,192,664–3,298,863**	106,199	p23.2	CN Loss	0.0	1	*CSMD1*
**chr8:5,543,815–5,742,245**	198,430	p23.2	CN Loss	100.0	0	
**chr8:6,820,292–9,022,725**	2,202,433	p23.1	CN Loss	68.7	59	*DEFA1, DEFA1B, DEFT1P2, DEFT1P, DEFA1, DEFA1B, DEFT1P2, DEFT1P, DEFA3, DEFA11P, DEFA5, LOC349196, LOC349196, LOC349196, DEFB109P1B , FAM66B, USP17L1P, USP17L4, ZNF705G, DEFB4B, DEFB103B, DEFB103A, SPAG11B, DEFB104A, DEFB104B, DEFB106A,*

						DEFB106B, DEFB105A, DEFB105B, DEFB107A, DEFB107B, FAM90A7P, FAM90A7P, FAM90A10P, DEFB107A, DEFB107B, DEFB105A, DEFB105B, DEFB106A, DEFB106B, DEFB104A, DEFB104B, SPAG11B, SPAG11A, DEFB103A, DEFB103B, DEFB4A, ZNF705B, USP17L8, USP17L3, FAM66E, DEFB109P1B , MIR548I3, FAM86B3P, SGK223, CLDN23, MFHAS1, ERI1, MIR4660
**chr8:9,108,088–9,506,118**	398,030	p23.1	CN Loss	0.3	2	*LOC157273, TNKS*
**chr8:9,699,935–9,786,655**	86,720	p23.1	CN Loss	0.0	0	
**chr8:12,480,144–12,641,126**	160,982	p23.1	CN Loss	100.0	4	*LOC729732, MIR3926–1, MIR3926–2, LONRF1*
**chr8:14,144,737–15,008,775**	864,038	p22	CN Loss	1.8	2	*SGCZ, MIR383*
**chr8:17,753,137–17,933,149**	180,012	p22	CN Loss	0.0	2	*FGL1, PCM1*
**chr8:18,105,847–**	241,893	p22	CN	0.0	2	*NAT1, NAT2*
**18,347,740**			Loss			
**chr8:19,940,444–20,004,254**	63,810	p21.3	CN Loss	0.0	0	
**chr8:21,810,399–21,947,727**	137,328	p21.3	CN Loss	0.0	3	*DOK2, XPO7, NPM2*
**chr8:22,101,084–23,436,255**	1,335,171	p21.3-p21.2	CN Loss	1.3	28	*BMP1, PHYHIP, MIR320A, POLR3D, PIWIL2, SLC39A14, PPP3CC, SORBS3, PDLIM2, C8orf58, KIAA1967, FLJ14107, BIN3, EGR3, PEBP4, RHOBTB2, TNFRSF10B, LOC286059, LOC254896, TNFRSF10C, TNFRSF10D, TNFRSF10A, LOC389641, CHMP7, R3HCC1, LOXL2, LOC10050715 6, ENTPD4*
**chr8:23,774,569–24,321,211**	546,642	p21.2	CN Loss	3.1	2	*ADAM28, ADAMDEC1*
**chr8:25,872,199–26,078,696**	206,497	p21.2	CN Loss	0.0	1	*EBF2*
**chr8:26,337,410–28,069,477**	1,732,067	p21.2-p21.1	CN Loss	0.4	19	*PNMA2, DPYSL2, ADRA1A, STMN4, TRIM35, PTK2B, CHRNA2, EPHX2, CLU, SCARA3, MIR3622A,*
						MIR3622B, CCDC25, ESCO2, PBK, MIR4287, SCARA5, NUGGC, ELP3
**chr8:28,135,907–28,195,087**	59,180	p21.1	CN Loss	0.0	0	
**chr8:30,398,928–30,577,619**	178,691	p12	CN Loss	0.0	2	*RBPMS, GTF2E2*
**chr8:37,261,348–37,414,728**	153,380	p12	CN Loss	0.0	0	
**chr8:37,553,512–38,049,159**	495,647	p12	CN Loss	0.0	10	*ZNF703, ERLIN2, LOC728024, PROSC, GPR124, BRF2, RAB11FIP1, GOT1L1, ADRB3, EIF4EBP1*
**chr8:39,252,098–39,337,749**	85,651	p11.23	CN Loss	51.7	2	*ADAM32, ADAM5*
**chr8:39,508,473–39,628,658**	120,185	p11.22	CN Loss	57.5	2	*LOC10013096 4, ADAM18*
**chr9:1,934,152–3,256,829**	1,322,677	p24.3-p24.2	CN Gain	0.1	6	*SMARCA2, FLJ35024, VLDLR, KCNV2, KIAA0020, RFX3*
**chr9:3,272,788–10,248,806**	6,976,018	p24.2-p23	CN Gain	1.2	31	*RFX3, GLIS3–AS1, GLIS3, SLC1A1, SPATA6L, PPAPDC2, CDC37L1, AK3, RCL1, MIR101–2, JAK2, INSL6, INSL4, RLN2, RLN1, PLGRKT,*
						CD274, PDCD1LG2, KIAA1432, ERMP1, MLANA, KIAA2026, MIR4665, RANBP6, IL33, TPD52L3, UHRF2, GLDC, KDM4C, C9orf123, PTPRD
**chr9:10,761,919–12,567,760**	1,805,841	p23	CN Gain	19.9	0	
**chr9:13,084,687–13,177,901**	93,214	p23	CN Gain	0.0	1	*MPDZ*
**chr9:13,244,498–14,505,317**	1,260,819	p23-p22.3	CN Gain	0.0	4	*MPDZ, FLJ41200, LINC00583,*

						NFIB
**chr9:16,475,837–17,181,075**	705,238	p22.3-p22.2	CN Gain	0.0	2	*BNC2, CNTLN*
**chr9:19,031,830–19,044,266**	12,436	p22.1	CN Gain	0.0	2	*RRAGA, HAUS6*
**chr9:20,612,727–21,131,395**	518,668	p21.3	CN Gain	0.6	5	*MIR491, FOCAD, PTPLAD2, IFNB1, IFNW1*
**chr9:21,328,058–21,907,688**	579,630	p21.3	CN Gain	0.0	9	*IFNA6, IFNA13, IFNA2, IFNA8, IFNA1, IFNE, MIR31HG, MIR31, MTAP*
**chr9:23,263,046–23,717,938**	454,892	p21.3	CN Gain	3.3	1	*ELAVL2*
**chr9:25,505,935–25,682,015**	176,080	p21.2	CN Gain	0.0	1	*TUSC1*
**chr9:32,501,115–33,201,863**	700,748	p21.1-p13.3	CN Gain	0.7	10	*DDX58, TOPORS, LOC10012925 0, NDUFB6, TAF1L, TMEM215, APTX, DNAJA1, SMU1, B4GALT1*
**chr9:33,262,141–33,357,824**	95,683	p13.3	CN Gain	0.0	2	*CHMP5, NFX1*
**chr9:33,415,054–33,460,111**	45,057	p13.3	CN Gain	0.0	2	*AQP3, NOL6*
**chr9:33,580,807–33,694,356**	113,549	p13.3	CN Gain	0.0	2	*ANXA2P2, PTENP1*
**chr9:33,912,931–34,065,134**	152,203	p13.3	CN Gain	0.0	3	*SNORD121B, SNORD121A, UBAP2*
**chr9:37,803,369–38,050,915**	247,546	p13.2	CN Gain	0.0	3	*DCAF10, SLC25A51, SHB*
**chr9:86,776,995–86,785,994**	8999	q21.33	CN Loss	0.0	0	
**chr9:118,108,362–119,065,161**	956,799	q33.1	CN Loss	0.0	1	*DBC1*
**chr9:131,381,496–131,526,405**	144,909	q34.13	CN Loss	0.0	8	*PRRC2B, SNORD62A, SNORD62B, SNORD62A, SNORD62B, POMT1, UCK1, RAPGEF1*
**chr9:132,164,868–132,207,419**	42,551	q34.13	CN Loss	0.0	1	*SETX*
**chr9:132,379,055–132,484,506**	105,451	q34.13	CN Loss	0.0	1	*C9orf171*
**chr9:133,330,358–133,379,264**	48,906	q34.2	CN Loss	0.0	3	*ADAMTS13, CACFD1, SLC2A6*
**chr9:133,659,673–133,743,260**	83,587	q34.2	CN Loss	0.0	1	*VAV2*
**chr9:133,977,094–134,047,962**	70,868	q34.2	CN Loss	0.0	1	*WDR5*
**chr9:137,815,000 –137,822,872**	7872	q34.3	CN Loss	0.0	1	*EHMT1*
**chr11:48,338,680 –49,035,816**	697,136	p11.2– p11.12	CN Loss	1.6	4	*OR4A47, TRIM51GP, TRIM49B, TRIM64C*
**chr11:54,789,837–54,920,601**	130,764	q11	CN Loss	2.5	4	*TRIM48, TRIM51HP, OR4A16, OR4A15*
**chr11:54,984,140–55,028,017**	43,877	q11	CN Loss	0.0	0	
**chr11:55,234,512–55,245,355**	10,843	q11	CN Loss	100.0	0	
**chr11:55,430,801–55,440,341**	9540	q11	CN Loss	0.0	1	*OR5W2*
**chr11:79,573,296–80,192,881**	619,585	q14.1	CN Loss	7.8	0	
**chr11:114,705,32 4–114,770,907**	65,583	q23.3	CN Loss	0.0	1	*CADM1*
**chr11:121,200,53 5–121,256,571**	56,036	q24.1	CN Loss	0.0	0	
**chr11:123,555,00 9–123,596,823**	41,814	q24.1	CN Loss	0.0	0	
**chr11:123,762,61 9–123,857,511**	94,892	q24.1-q24.2	CN Loss	0.0	3	*OR8B3, OR8B4, OR8B8*
**chr11:124,111,73 0–124,524,064**	412,334	q24.2	CN Loss	0.0	11	*NRGN, VSIG2, ESAM, MSANTD2, ROBO3, ROBO4, HEPN1, HEPACAM, CCDC15, SLC37A2, TMEM218*
**chr11:124,791,56 5–124,811,913**	20,348	q24.2	CN Loss	0.0	1	*PKNOX2*
**chr11:125,688,45 4–125,871,027**	182,573	q24.2	CN Loss	0.0	4	*DCPS, FLJ39051, ST3GAL4, KIRREL3*
**chr11:126,652,65 3–126,922,796**	270,143	q24.2	CN Loss	0.0	0	
**chr11:128,066,08 3–128,285,672**	219,589	q24.3	CN Loss	0.0	5	*FLI1–AS1, FLI1, KCNJ1, C11orf45, KCNJ5*
**chr11:129,792,32 0–130,252,874**	460,554	q24.3	CN Loss	0.0	4	*ADAMTS8, ADAMTS15, C11orf44, SNX19*
**chr11:133,410,35 9–133,657,612**	247,253	q25	CN Loss	0.0	7	*LOC10012823 9, JAM3, NCAPD3, VPS26B, THYN1, ACAD8, GLB1L3*

**chr13:18,065,953–18,419,347**	353,394	q11-q12.11	CN Gain	82.8	1	***ANKRD20A9 P***
**chr13:20,059,415–20,431,085**	371,670	q12.11	CN Gain	0.0	4	***IFT88, IL17D, N6AMT2, XPO4***
**chr13:20,926,840–20,932,672**	5832	q12.11	CN Gain	0.0	1	***ZDHHC20***
**chr13:46,771,673–47,079,768**	308,095	q14.2	CN Gain	0.0	0	
**chr13:49,862,191–49,941,428**	79,237	q14.3	CN Gain	0.0	0	
**chr13:50,241,792–50,451,595**	209,803	q14.3	CN Gain	0.3	4	***DLEU7, DLEU7–AS1, RNASEH2B–AS1, RNASEH2B***
**chr13:51,111,933–51,209,836**	97,903	q14.3	CN Gain	0.0	1	***WDFY2***
**chr13:63,584,603–64,531,695**	947,092	q21.31	CN Gain	0.0	0	
**chr13:65,213,613–65,821,403**	607,790	q21.32	CN Gain	0.0	3	***MIR548X2, MIR4704, PCDH9***
**chr13:66,027,749–66,058,947**	31,198	q21.32	CN Gain	100.0	1	***PCDH9***
**chr13:66,093,042–66,156,159**	63,117	q21.32	CN Gain	0.0	1	***PCDH9***
**chr13:66,511,486–66,924,124**	412,638	q21.32	CN Gain	0.0	1	***PCDH9***
**chr13:67,365,515**	180,209	q21.32–	CN	0.0	0	
**–67,545,724**		q21.33	Gain			
**chr13:73,519,926–73,591,864**	71,938	q22.1	CN Gain	0.0	1	***KLF12***
**chr13:77,091,913–77,120,617**	28,704	q22.3	CN Gain	0.0	1	***SCEL***
**chr13:79,263,868–79,425,040**	161,172	q31.1	CN Gain	0.0	0	
**chr13:80,128,565–80,449,023**	320,458	q31.1	CN Gain	0.0	0	
**chr13:107,027,908–107,048,590**	20,682	q33.3	CN Gain	0.0	1	***FAM155A***
**chr13:107,967,995–108,597,339**	629,344	q33.3	CN Gain	0.0	1	***MYO16***
**chr13:110,020,406–110,544,514**	524,108	q34	CN Gain	0.0	5	***CARKD, CARS2, ING1, LINC00346, ANKRD10***
**chr13:110,557,042–111,966,665**	1,409,623	q34	CN Gain	0.0	3	***ARHGEF7, TEX29, SOX1***
**chr13:112,502,030–112,673,447**	171,417	q34	CN Gain	0.0	3	***ATP11A, MCF2L–AS1, MCF2L***
**chr13:112,720,269–112,849,806**	129,537	q34	CN Gain	0.0	3	***MCF2L, F7, F10***
**chr13:112,913,326–113,004,883**	91,557	q34	CN Gain	0.0	2	***CUL4A, LAMP1***
**chr13:113,220,267–114,142,980**	922,713	q34	CN Gain	0.0	13	***TMCO3, TFDP1, ATP4B, GRK1, LINC00565, LOC10050639 4, GAS6, GAS6-AS1, TMEM255B, RASA3, CDC16, UPF3A, CHAMP1***
**chr14:19,808,051–19,879,596**	71,545	q11.2	CN Loss	0.0	3	***TTC5, CCNB1IP1, SNORD126***
**chr14:19,942,006–20,534,688**	592,682	q11.2	CN Loss	10.1	21	***TEP1, KLHL33, OSGEP, APEX1, TMEM55B, PNP, RNASE10, RNASE9, RNASE11, RNASE12, OR6S1, ANG, RNASE4, EDDM3A, EDDM3B, RNASE6, RNASE1, RNASE3, ECRP, RNASE2, METTL17***
**chr14:21,131,506–21,157,261**	25,755	q11.2	CN Loss	0.0	0	
**chr14:21,409,247–21,434,637**	25,390	q11.2	CN Loss	100.0	0	
**chr14:21,671,956–21,743,735**	71,779	q11.2	CN Loss	100.0	0	
**chr14:22,465,578–22,607,869**	142,291	q11.2	CN Loss	0.0	9	***PRMT5, HAUS4, MIR4707, AJUBA, C14orf93, PSMB5, PSMB11, CDH24, ACIN1***
**chr14:22,615,515–22,632,327**	16,812	q11.2	CN Loss	0.0	1	***ACIN1***
**chr14:23,677,922–23,751,956**	74,034	q11.2	CN Loss	0.0	10	***PSME1, EMC9, PSME2, RNF31, IRF9, REC8, IPO4, TM9SF1, TSSK4, CHMP4A***
**chr14:24,456,066**	443,215	q12	CN	0.3	1	***STXBP6***
**–24,899,281**			Loss			
**chr14:29,101,429–29,234,859**	133,430	q12	CN Loss	0.0	1	***PRKD1***
**chr14:29,885,479–30,068,871**	183,392	q12	CN Loss	0.0	0	
**chr14:30,270,202–30,293,223**	23,021	q12	CN Loss	0.0	1	***SCFD1***
**chr14:30,430,997–30,640,692**	209,695	q12	CN Loss	0.0	4	***STRN3, MIR624, AP4S1, HECTD1***
**chr14:31,100,236–31,198,516**	98,280	q12	CN Loss	0.0	2	***RNU6-16, NUBPL***
**chr14:31,893,257–32,079,668**	186,411	q12	CN Loss	0.0	1	***AKAP6***
**chr14:32,170,549–32,341,433**	170,884	q12	CN Loss	0.0	1	***AKAP6***
**chr14:33,255,411–33,507,210**	251,799	q13.1	CN Loss	0.0	2	***NPAS3, EGLN3***
**chr14:34,699,297–34,951,855**	252,558	q13.2	CN Loss	0.0	3	***KIAA0391, PSMA6, NFKBIA***

**chr14:35,089,377–35,367,707**	278,330	q13.2	CN Loss	0.0	2	*RALGAPA1, BRMS1L*
**chr14:35,556,347–35,661,337**	104,990	q13.2-q13.3	CN Loss	0.0	1	*LINC00609*
**chr14:35,682,484–35,813,344**	130,860	q13.3	CN Loss	2.3	2	*LINC00609, PTCSC3*
**chr14:36,063,641–36,078,282**	14,641	q13.3	CN Loss	0.0	0	
**chr14:37,021,167–37,079,803**	58,636	q21.1	CN Loss	0.0	1	*MIPOL1*
**chr14:37,839,846–37,974,393**	134,547	q21.1	CN Loss	0.0	0	
**chr14:45,475,327–45,600,580**	125,253	q21.2	CN Loss	0.0	0	
**chr14:47,354,464–48,020,135**	665,671	q21.3	CN Loss	1.5	0	
**chr14:50,212,681–50,221,394**	8713	q22.1	CN Loss	0.0	0	
**chr14:58,159,179–58,169,617**	10,438	q23.1	CN Loss	0.0	0	
**chr14:64,025,225–64,066,957**	41,732	q23.3	CN Loss	0.0	2	*ZBTB25, ZBTB1*
**chr14:64,468,309–64,616,437**	148,128	q23.3	CN Loss	0.0	7	*CHURC1, GPX2, RAB15, CHURC1-FNTB, FNTB, MIR4706, MAX*
**chr14:65,216,309–65,227,085**	10,776	q23.3	CN Loss	0.0	1	*FUT8*
**chr14:66,289,598–66,343,213**	53,615	q23.3	CN Loss	0.0	1	*GPHN*
**chr14:68,070,078–68,078,261**	8183	q24.1	CN Loss	0.0	1	*RAD51B*
**chr14:68,147,384–68,316,581**	169,197	q24.1	CN Loss	0.0	0	
**chr14:75,010,936–75,190,330**	179,394	q24.3	CN Loss	0.0	3	*BATF, FLVCR2, C14orf1*
**chr14:76,921,429–76,926,634**	5205	q24.3	CN Loss	0.0	1	*SAMD15*
**chr14:77,054,665–77,298,258**	243,593	q24.3	CN Loss	0.0	5	*SPTLC2, ALKBH1, SLIRP, SNW1, C14orf178*
**chr14:77,302,965–77,315,530**	12,565	q24.3	CN Loss	0.0	1	*C14orf178*
**chr14:85,385,179–85,506,646**	121,467	q31.3	CN Loss	0.0	0	
**chr14:85,650,613–86,446,911**	796,298	q31.3	CN Loss	0.0	1	*LOC283585*
**chr14:88,542,999–88,563,278**	20,279	q32.11	CN Loss	0.0	0	
**chr14:90,172,729–90,532,073**	359,344	q32.12	CN Loss	0.0	2	*TTC7B, RPS6KA5*
**chr14:90,719,732–90,775,532**	55,800	q32.12	CN Loss	0.0	2	*C14orf159, GPR68*
**chr14:90,826,366–90,877,803**	51,437	q32.12	CN Loss	13.7	1	*CCDC88C*
**chr14:91,160,620–91,628,513**	467,893	q32.12	CN Loss	0.0	5	*CATSPERB, TC2N, FBLN5, TRIP11, ATXN3*
**chr14:91,920,394**	406,473	q32.12	CN	0.0	3	*SLC24A4,*
**–92,326,867**			Loss			*RIN3, LGMN*
**chr14:95,850,445–95,864,146**	13,701	q32.2	CN Loss	0.0	1	*ATG2B*
**chr14:100,622,84 2–101,449,140**	826,298	q32.31	CN Loss	1.8	5	*DIO3OS, MIR1247, DIO3, LINC00239, PPP2R5C*
**chr14:101,497,57 7–101,582,372**	84,795	q32.31-q32.32	CN Loss	0.0	1	*DYNC1H1*
**chr14:101,831,52 6–101,956,883**	125,357	q32.32	CN Loss	0.0	4	*MOK, ZNF839, CINP, TECPR2*
**chr14:102,197,70 8–102,372,289**	174,581	q32.32	CN Loss	0.0	2	*RCOR1, TRAF3*
**chr14:102,514,37 8–102,635,142**	120,764	q32.32	CN Loss	0.0	1	*CDC42BPB*
**chr14:102,871,84 8–102,931,032**	59,184	q32.32	CN Loss	0.0	3	*SNORA28, EIF5, MARK3*
**chr14:104,270,02 1–104,273,557**	3536	q32.33	CN Loss	0.0	1	*ADSSL1*
**chr14:106,172,52 6–106,368,585**	196,059	q32.33	CN Loss	74.3	0	
**chr15:39,669,823–39,721,803**	51,980	q15.1	CN Loss	0.0	0	
**chr15:46,719,007–46,741,098**	22,091	q21.1	CN Loss	0.0	1	*FBN1*
**chr15:47,748,774–48,210,910**	462,136	q21.2	CN Loss	0.0	1	*ATP8B4*
**chr15:50,188,307–50,544,741**	356,434	q21.2	CN Loss	0.0	5	*BCL2L10, GNB5, MYO5C, MIR1266, MYO5A*
**chr15:50,826,333–50,843,466**	17,133	q21.3	CN Loss	0.0	1	*HNF6*
**chr15:51,605,915–51,756,728**	150,813	q21.3	CN Loss	0.0	1	*WDR72*
**chr15:53,065,652–53,417,306**	351,654	q21.3	CN Loss	0.0	3	*RSL24D1, RAB27A, PIGB*
**chr15:53,427,831–53,604,156**	176,325	q21.3	CN Loss	0.0	6	*PIGB, MIR628, CCPG1,*
						FLJ27352, DYX1C1-CCPG1, DYX1C1
**chr15:53,689,271–53,904,157**	214,886	q21.3	CN Loss	0.0	1	*PRTG*
**chr15:54,039,484–54,061,026**	21,542	q21.3	CN Loss	0.0	1	*NEDD4*
**chr15:54,320,234–54,407,485**	87,251	q21.3	CN Loss	0.0	1	*RFX7*
**chr15:55,449,459–56,222,552**	773,093	q21.3	CN Loss	0.0	6	*CGNL1, MYZAP, GCOM1, POLR2M, ALDH1A2, AQP9*
**chr15:56,630,587–56,734,190**	103,603	q21.3	CN Loss	0.0	2	*LIPC, ADAM10*

**chr15:56,854,439–56,923,398**	68,959	q21.3	CN Loss	0.0	1	*FAM63B*
**chr15:61,448,730–61,478,020**	29,290	q22.2	CN Loss	0.0	1	*CA12*
**chr15:62,990,918–63,023,391**	32,473	q22.31	CN Loss	0.0	1	*ANKDD1A*
**chr15:63,129,167–63,186,496**	57,329	q22.31	CN Loss	0.0	4	*SLC51B, RASL12, KBTBD13, UBAP1L*
**chr15:63,570,638–63,794,541**	223,903	q22.31	CN Loss	0.0	5	*DPP8, PTPLAD1, VWA9, SLC24A1, DENND4A*
**chr15:69,922,996–69,937,813**	14,817	q23	CN Loss	0.0	1	*MYO9A*
**chr15:72,526,739–72,648,021**	121,282	q24.1	CN Loss	0.0	3	*UBL7, LOC440288, ARID3B*
**chr15:72,702,022–72,840,230**	138,208	q24.1	CN Loss	0.0	4	*CLK3, EDC3, CYP1A1, CYP1A2*
**chr15:73,091,745–73,416,859**	325,114	q24.1-q24.2	CN Loss	0.0	6	*SCAMP5, PPCDC, C15orf39, GOLGA6C, GOLGA6D,*
						COMMD4
**chr15:73,701,548–73,821,706**	120,158	q24.2	CN Loss	0.0	6	*SNUPN, IMP3, SNX33, CSPG4, ODF3L1, DNM1P35*
**chr15:75,048,739–75,510,213**	461,474	q24.3	CN Loss	0.0	5	*PSTPIP1, TSPAN3, LINC00597, PEAK1, HMG20A*
**chr15:76,072,953–76,283,942**	210,989	q24.3-q25.1	CN Loss	0.0	6	*LOC91450, TBC1D2B, SH2D7, CIB2, IDH3A, ACSBG1*
**chr15:79,413,377–79,473,916**	60,539	q25.1	CN Loss	0.0	1	*TMC3*
**chr15:81,452,388–81,533,579**	81,191	q25.2	CN Loss	0.0	3	*C15orf40, BTBD1, MIR4515*
**chr15:82,505,738–83,278,235**	772,497	q25.2-q25.3	CN Loss	38.4	19	*EFTUD1P1, DNM1P41, LOC10050567 9, LOC642423, LOC440300, LOC388152, GOLGA6L4, DNM1P41, GOLGA6L5, UBE2Q2P1, LOC10050687 4, ZSCAN2, SCAND2, WDR73, NMB, SEC11A, ZNF592, ALPK3, SLC28A1*
**chr15:83,445,433–83,804,111**	358,678	q25.3	CN Loss	2.1	3	*PDE8A, LOC642423, AKAP13*
**chr15:83,940,783–84,081,736**	140,953	q25.3	CN Loss	0.0	1	*AKAP13*
**chr15:87,518,064–87,849,590**	331,526	q26.1	CN Loss	4.9	8	*ABHD2, RLBP1, FANCI, POLG, MIR9–3, LOC254559, RHCG, LOC283761*
**chr15:88,026,032–88,052,390**	26,358	q26.1	CN Loss	0.0	2	*PEX11A, WDR93*
**chr15:88,120,990–88,413,815**	292,825	q26.1	CN Loss	1.5	7	*MESP2, ANPEP, MIR5094, AP3S2, C15orf38–AP3S2, C15orf38, ZNF710*
**chr15:88,634,497–88,759,698**	125,201	q26.1	CN Loss	0.0	3	*GABARAPL3, ZNF774, IQGAP1*
**chr15:90,847,328–91,360,624**	513,296	q26.1	CN Loss	0.0	6	*LOC10014460 4, FAM174B, ASB9P1, LOC10050721 7, MIR3175, CHD2*
**chr15:98,023,402–98,179,732**	156,330	q26.3	CN Loss	0.0	3	*MEF2A, LYSMD4, DNM1P46*
**chr17:5,526,400–5,588,767**	62,367	p13.2	CN Loss	0.0	0	
**chr17:21,431,559–21,674,159**	242,600	p11.2	CN Loss	11.6	0	
**chr17:24,083,391–24,314,380**	230,989	q11.2	CN Gain	0.0	12	*NEK8, TRAF4, FAM222B, ERAL1, MIR451A, MIR451B, MIR144, MIR4732, FLOT2, DHRS13,*
						PHF12, SEZ6
**chr17:25,066,969–25,138,469**	71,500	q11.2	CN Gain	0.0	1	*SSH2*
**chr17:32,784,580–33,098,353**	313,773	q12	CN Gain	0.7	6	*ACACA, C17orf78, TADA2A, DUSP14, SYNRG, DDX52*
**chr17:38,570,907–38,829,651**	258,744	q21.31	CN Gain	17.3	4	*NBR1, TMEM106A, TMEM106A–AS1, LOC10013058 1*
**chr17:54,059,332–54,748,763**	689,431	q23.2	CN Gain	0.2	10	*TEX14, RAD51C, PPM1E, TRIM37, SKA2, MIR454, MIR301A, PRR11, SMG8, GDPD1*
**chr17:54,969,023–55,120,848**	151,825	q23.2	CN Gain	0.0	2	*DHX40, CLTC*
**chr17:55,126,726–55,133,381**	6655	q23.2	CN Gain	0.0	2	*CLTC, PTRH2*
**chr17:55,661,447–55,682,369**	20,922	q23.2	CN Gain	0.0	2	*SCARNA20, USP32*
**chr17:57,286,642–57,474,557**	187,915	q23.2	CN Gain	0.0	3	*BRIP1, INTS2, MED13*
**chr17:57,494,180–58,081,012**	586,832	q23.2-q23.3	CN Gain	0.0	6	*MED13, TBC1D3P2, EFCAB3, METTL2A, TLK2, MRC2*
**chr17:62,469,882–62,632,674**	162,792	q24.2	CN Gain	0.0	2	*CACNG1, HELZ*

**chr17:62,823,228–62,982,920**	159,692	q24.2	CN Gain	0.0	2	*MIR548AA2, PITPNC1*
**chr17:72,252,138–72,289,494**	37,356	q25.1	CN Gain	0.0	1	*MFSD11*
**chr18:142,250–471,404**	329,154	p11.32	CN Loss	3.3	3	*USP14, THOC1, COLEC12*
**chr18:818,102–825,963**	7861	p11.32	CN Loss	0.0	0	
**chr18:1,411,976–1,469,679**	57,703	p11.32	CN Loss	0.0	0	
**chr18:2,923,913–3,023,053**	99,140	p11.31	CN Loss	0.0	2	*LOC727896, LPIN2*
**chr18:3,931,287–4,504,523**	573,236	p11.31	CN Loss	1.3	2	*DLGAP1, DLGAP1–AS5*
**chr18:4,642,369–5,282,511**	640,142	p11.31	CN Loss	0.0	4	*C18orf42, LINC00526, LINC00667, ZFP161*
**chr18:9,884,667–10,054,928**	170,261	p11.22	CN Loss	0.0	1	*VAPA*
**chr18:11,984,291–12,302,067**	317,776	p11.21	CN Loss	0.0	4	*IMPA2, C18orf61, CIDEA, TUBB6*
**chr18:12,430,766–12,673,891**	243,125	p11.21	CN Loss	0.0	3	*SPIRE1, CEP76, PSMG2*
**chr18:12,691,989–14,091,725**	1,399,736	p11.21	CN Loss	0.0	14	*CEP76, PSMG2, PTPN2, SEH1L, CEP192, LOC10028812 2, LDLRAD4, MIR5190, MIR4526, FAM210A, RNMT, MC5R, MC2R, ZNF519*
**chr18:17,691,079–18,004,067**	312,988	q11.2	CN Loss	0.3	2	*MIB1, GATA6*
**chr18:19,195,122–19,361,994**	166,872	q11.2	CN Loss	0.0	3	*TMEM241, RIOK3, C18orf8*
**chr18:21,213,576**	496,304	q11.2	CN	0.0	0	
**–21,709,880**			Loss			
**chr18:22,931,625–22,955,852**	24,227	q11.2	CN Loss	0.0	1	*CHST9*
**chr18:24,103,460–24,447,720**	344,260	q12.1	CN Loss	0.0	0	
**chr18:25,528,591–26,129,267**	600,676	q12.1	CN Loss	0.9	0	
**chr18:27,851,691–27,921,721**	70,030	q12.1	CN Loss	0.0	1	*RNF125*
**chr18:29,012,906–29,275,546**	262,640	q12.1	CN Loss	0.0	1	*CCDC178*
**chr18:29,473,060–29,736,659**	263,599	q12.1	CN Loss	0.0	2	*ASXL3, NOL4*
**chr18:29,808,480–30,154,768**	346,288	q12.1	CN Loss	0.0	1	*NOL4*
**chr18:30,431,568–31,838,877**	1,407,309	q12.1-q12.2	CN Loss	0.0	13	*DTNA, MAPRE2, ZNF397, ZSCAN30, ZNF271, ZNF24, ZNF396, INO80C, MIR3975, GALNT1, MIR187, C18orf21, RPRD1A*
**chr18:33,386,018–33,707,865**	321,847	q12.2	CN Loss	0.0	2	*CELF4, MIR4318*
**chr18:34,475,739–34,493,818**	18,079	q12.2	CN Loss	0.0	0	
**chr18:38,391,943–38,631,170**	239,227	q12.3	CN Loss	0.0	2	*LOC284260, RIT2*
**chr18:41,954,284–41,981,261**	26,977	q21.1	CN Loss	0.0	1	*HAUS1*
**chr18:42,549,128–42,633,040**	83,912	q21.1	CN Loss	0.0	1	*ST8SIA5*
**chr20:85,564–168,766**	83,202	p13	CN Loss	0.0	3	*DEFB127, DEFB128, DEFB129*
**chr20:604,610–1,511,702**	907,092	p13	CN Loss	0.9	18	*SCRT2, SLC52A3, FAM110A, ANGPT4, RSPO4, PSMF1, TMEM74B, C20orf202, RAD21L1, SNPH, SDCBP2, FKBP1A–SDCBP2, SDCBP2–AS1, FKBP1A, NSFL1C, SIRPB2, SIRPD, SIRPB1*
**chr20:1,543,029–2,278,494**	735,465	p13	CN Loss	0.5	7	*SIRPB1, SIRPG, LOC10028947 3, SIRPA, PDYN, STK35, TGM3*
**chr20:4,163,287–4,643,781**	480,494	p13	CN Loss	1.2	2	*ADRA1D, PRNP*
**chr20:4,836,072–5,158,170**	322,098	p13-p12.3	CN Loss	0.0	5	*SLC23A2, TMEM230, PCNA-AS1, PCNA, CDS2*
**chr20:5,748,138–7,167,999**	1,419,861	p12.3	CN Loss	0.0	8	*C20orf196, CHGB, TRMT6, MCM8, CRLS1, LRRN4, FERMT1, BMP2*
**chr20:7,910,186–12,535,405**	4,625,219	p12.3-p12.1	CN Loss	0.2	13	*TMX4, PLCB1, PLCB4, LAMP5, PAK7, ANKRD5, SNAP25-AS1,*
						SNAP25, MKKS, SLX4IP, JAG1, LOC339593, BTBD3
**chr20:14,002,958–14,119,675**	116,717	p12.1	CN Loss	0.0	1	*MACROD2*
**chr20:27,100,000–28,266,172**	1,166,172	q11.1	CN Gain	7.3	3	*FRG1B, LOC642236, MLLT10P1*
**chr20:38,914,556–39,667,021**	752,465	q12	CN Gain	1.2	6	*TOP1, PLCG1, ZHX3, LPIN3, EMILIN3, CHD6*
**chr20:39,924,701–40,217,630**	292,929	q12	CN Gain	0.0	1	*PTPRT*
**chr20:43,182,807–43,715,277**	532,470	q13.12	CN Gain	0.0	22	*WFDC12, PI3, SEMG1, SEMG2, SLPI, MATN4, RBPJL, SDC4, SYS1, TP53TG5, SYS1-DBNDD2, DBNDD2, PIGT, WFDC2,*

						SPINT3, WFDC6, EPPIN-WFDC6, EPPIN, WFDC8, WFDC9, WFDC10A, WFDC11
**chr20:49,017,282–49,536,308**	519,026	q13.13-q13.2	CN Gain	0.0	3	*KCNG1, MIR3194, NFATC2*
**chr20:52,091,103–52,625,110**	534,007	q13.2	CN Gain	0.6	5	*BCAS1, MIR4756,*
						CYP24A1, PFDN4, DOK5
**chr20:55,932,323–56,527,968**	595,645	q13.32	CN Gain	0.0	6	*C20orf85, PPP4R1L, RAB22A, VAPB, APCDD1L, APCDD1L-AS1*
**chr20:57,616,783–57,848,527**	231,744	q13.33	CN Gain	0.0	2	*LOC10050638 4, PHACTR3*
**chr20:60,580,608–61,681,007**	1,100,399	q13.33	CN Gain	4.3	35	*SLCO4A1, LOC10012788 8, NTSR1, LINC00659, MRGBP, OGFR, COL9A3, DPH3P1, TCFL5, DIDO1, GID8, SLC17A9, BHLHE23, LINC00029, LOC10014459 7, LOC63930, HAR1B, HAR1A, MIR124-3, YTHDF1, BIRC7, MIR3196, NKAIN4, FLJ16779, ARFGAP1, MIR4326, COL20A1, CHRNA4, KCNQ2, EEF1A2, PPDPF, PTK6, SRMS, C20orf195, HELZ2*
**chr20:62,049,989–62,100,343**	50,354	q13.33	CN Gain	0.0	5	*UCKL1, UCKL1-AS1, ZNF512B, SAMD10, PRPF6*
**chr22:17,753,414–17,894,591**	141,177	q11.21	CN Loss	0.0	6	*HIRA, MRPL40, C22orf39, UFD1L, CDC45, CLDN5*
**chr22:18,008,241–18,116,460**	108,219	q11.21	CN Loss	0.0	3	*SEPT5, SEPT5-GP1BB, GP1BB*
**chr22:18,379,998–18,446,807**	66,809	q11.21	CN Loss	0.0	3	*MIR185, TANGO2, DGCR8*
**chr22:19,700,181–19,726,957**	26,776	q11.21	CN Loss	0.0	4	*P2RX6, SLC7A4, P2RX6P, LOC400891*
**chr22:20,471,450–20,573,268**	101,818	q11.21-q11.22	CN Loss	0.0	1	*MAPK1*
**chr22:20,926,471–21,344,777**	418,306	q11.22	CN Loss	100.0	7	*LOC96610, ZNF280B, ZNF280A, PRAME, LOC648691, POM121L1P, GGTLC2*
**chr22:22,093,542–22,317,997**	224,455	q11.23	CN Loss	0.0	3	*IGLL1, C22orf43, GUSBP11*
**chr22:22,722,828–22,886,439**	163,611	q11.23	CN Loss	4.0	2	*GSTTP2, CABIN1*
**chr22:23,451,589–23,676,351**	224,762	q11.23	CN Loss	0.0	4	*PIWIL3, TOP1P2, SGSM1, TMEM211*
**chr22:24,975,375–26,236,804**	1,261,429	q12.1	CN Loss	0.0	10	*SEZ6L, ASPHD2, HPS4, SRRD, TFIP11, MIR548J, TPST2,*
						CRYBB1, CRYBA4, MIAT
**chr22:26,431,206–26,520,119**	88,913	q12.1	CN Loss	0.0	1	*MN1*
**chr22:27,750,436–27,757,126**	6690	q12.1	CN Loss	0.0	2	*ZNRF3, ZNRF3-AS1*
**chr22:28,572,986–28,769,738**	196,752	q12.2	CN Loss	0.0	1	*MTMR3*
**chr22:29,051,285–29,095,047**	43,762	q12.2	CN Loss	0.0	3	*SF3A1, CCDC157, KIAA1656*
**chr22:29,406,916–29,573,097**	166,181	q12.2	CN Loss	1.7	2	*MIR3200, OSBP2*
**chr22:31,004,821–32,262,860**	1,258,039	q12.3	CN Loss	0.2	10	*RFPL3, RFPL3-AS1, LOC339666, C22orf28, BPIFC, FBXO7, SYN3, TIMP3, MIR4764, LARGE*
**chr22:33,859,038–33,890,879**	31,841	q12.3	CN Loss	0.0	0	
**chr22:35,275,581–35,542,533**	266,952	q12.3	CN Loss	1.2	3	*CACNG2, IFT27, PVALB*
**chr22:37,563,348–37,657,804**	94,456	q13.1	CN Loss	2.9	2	*NPTXR, CBX6*
**chr22:37,735,270–37,795,940**	60,670	q13.1	CN Loss	0.0	3	*APOBEC3C, APOBEC3D, APOBEC3F*
**chr22:37,822,564–39,067,017**	1,244,453	q13.1	CN Loss	0.0	22	*APOBEC3H, CBX7, PDGFB, SNORD83B, SNORD83A, RPL3, RNU86, SNORD43, SYNGR1, TAB1, LOC10050647 2, MGAT3,*
						SMCR7L, ATF4, RPS19BP1, CACNA1I, ENTHD1, GRAP2, FAM83F, LOC10013089 9, TNRC6B, ADSL
**chr22:43,453,639–43,498,325**	44,686	q13.31	CN Loss	0.0	3	*PRR5, PRR5-ARHGAP8, ARHGAP8*
**chr22:44,249,179–44,256,822**	7643	q13.31	CN Loss	0.0	1	*FBLN1*
					1,099	

Table S11. Mann-Whitney (MW) *p*-values correlating metastasis-suppressor expression with CRC grade using a cohort composed of 1436 cases. *p*-Values highlighted in bold indicate significant correlation ($p > 0.05$).

Gene	Grade 1 *vs.* Grade 2. MW.pv	Grade 1 *vs.* Grade 3. MW.pv	Grade 2 *vs.* Grade 3. MW.pv
ADRA1A	0.200865	0.909476	0.07734
ADRA1D	0.102804	0.275505	0.477323
ADRB3	0.166421	**0.025572**	0.084262
APOBEC3D	0.833025	0.705971	0.457078
BRF2	0.108288	0.257709	0.640776
C20orf202	0.411843	0.784154	0.485192
TEX43	0.555174	0.616151	0.112187
CABIN1	0.512398	0.897197	0.351707
CACNA1I	0.230195	0.66045	0.480946
CSMD1	0.433949	0.080664	0.08836
DIO3	0.180268	0.121035	0.521137
EPHX2	0.951485	0.848353	0.823836
FAM83F	0.593739	0.756492	0.192038
GP1BB	**0.025659**	**0.016719**	0.390149
KIAA1656	**0.022059**	0.354918	0.33879
LOC339593	0.885246	0.702133	0.753526
MCM8	0.199375	0.590931	0.426505
NAT1	0.670187	0.538551	0.112849
NAT2	0.428978	0.29412	**0.001922**
HNF6	0.058524	0.201768	0.590868
PCDHGA11	0.113676	0.118576	0.620738
RAB11FIP1	0.53823	0.26208	0.417514
SPAG11A	0.067325	0.14933	0.754265
SIRPD	0.847406	0.72139	0.775775
TOP1P2	0.219921	0.872714	0.176931
WDR5	0.562789	0.491856	0.727828
ZNF366	0.108287	0.545399	0.392835
ZNF703	0.621442	0.069676	**0.011174**
ZNRF3	0.93572	0.062085	**0.006155**

Table S12. Mann-Whitney (MW) *p*-values correlating metastasis-suppressor expression with CRC stage using a cohort composed of 1436 cases. *p*-Values highlighted in bold indicate significant correlation ($p > 0.05$).

Gene	St. I *vs.* St. II	St. I *vs.* St. III	St. I *vs.* St. IV	St. II *vs.* St. III	St. II *vs.* St. IV	St. III *vs.* St. IV
ADRA1A	0.385629	0.054778	0.066347	0.127172	0.251446	0.849241
ADRA1D	0.785608	0.620774	0.116326	0.807158	**0.026466**	**0.047567**
ADRB3	0.10136	0.106675	**0.002672**	0.989223	**0.015087**	**0.014459**
APOBEC3D	0.712223	0.101441	0.153276	0.093201	0.193716	0.987492
BRF2	0.613642	0.415448	0.779541	0.486063	0.255382	0.108503
C20orf202	0.75567	0.462678	0.345101	0.526794	0.33763	0.677814
TEX43	0.318561	0.138558	0.279374	0.356343	0.767092	0.782308
CABIN1	0.544609	0.586498	0.779541	0.963063	0.161186	0.183319
CACNA1I	0.072997	0.493167	**0.028731**	0.139647	0.367745	**0.046696**
CSMD1	0.153858	0.042229	**0.007777**	0.311502	0.098403	0.448219
DIO3	**0.02618**	0.082522	**0.003979**	0.582364	0.32418	0.171977
EPHX2	0.065733	0.057956	0.18561	0.946155	0.631153	0.639529
FAM83F	0.8723	0.374491	0.226341	0.274343	0.125788	0.655018
GP1BB	0.275655	0.320917	0.050063	0.968447	0.092493	0.103424
KIAA1656	0.052232	0.081441	**0.01574**	0.828146	0.229776	0.222879
LOC339593	0.937582	0.21104	0.518774	**0.028447**	0.171109	0.771792
MCM8	0.62673	0.473988	0.83094	0.69029	0.726134	0.506484
NAT1	**0.023528**	**0.00892**	**0.01309**	0.251721	0.23828	0.682123
NAT2	**0.001433**	**0.000855**	**0.007777**	0.495168	0.990289	0.725755
HNF6	0.149155	0.208807	0.303232	0.859855	0.798256	0.943758
PCDHGA11	0.645843	0.455222	0.170335	0.68461	0.156389	0.288172
RAB11FIP1	0.461923	0.914349	0.118548	0.322231	0.120851	**0.039813**
SPAG11A	0.103964	0.117781	**0.018376**	0.908595	0.137917	0.123967
SIRPD	0.083448	0.052494	**0.000976**	0.425495	**0.020228**	0.103424
TOP1P2	0.486096	0.181304	0.060926	0.280316	0.08465	0.389628
WDR5	0.054005	0.071256	0.095858	0.693848	0.728164	0.890892
ZNF366	0.675705	0.885161	0.095857	0.597676	0.079882	**0.040961**
ZNF703	0.634052	0.356582	0.3801	0.288125	0.417887	0.866165
ZNRF3	0.687798	0.354983	0.212116	0.064105	0.196514	**0.00523**

Table S4. Mann-Whitney (MW) *p*-values correlating metastasis- suppressor expression with MSS *vs.* MSI CRC and Tumor (T) *vs.* Normal (N) using a cohort composed of 1436 cases.

Gene	MSS v. MSI-MW.pv	T v. N-MW.pv
ADRA1A	4.65×10^{-02}	0.000607
ADRA1D	6.67×10^{-02}	4.21×10^{-01}
ADRB3	0.018867	3.12×10^{-02}
APOBEC3D	0.538426	0.001437
BRF2	6.13×10^{-06}	1.44×10^{-01}
C20orf202	0.011137	5.24×10^{-02}
TEX43	4.67×10^{-03}	1.17×10^{-04}
CABIN1	4.10×10^{-08}	8.48×10^{-02}
CACNA1I	0.032276	4.77×10^{-05}
CSMD1	0.308815	7.95×10^{-01}
DIO3	4.58×10^{-05}	1.86×10^{-02}
EPHX2	2.40×10^{-01}	2.15×10^{-25}
FAM83F	0.047495	1.97×10^{-02}
GP1BB	4.17×10^{-02}	3.33×10^{-04}
KIAA1656	1.37×10^{-02}	1.53×10^{-04}
LOC339593	2.46×10^{-01}	8.75×10^{-01}
MCM8	2.74×10^{-02}	1.27×10^{-15}
NAT1	5.71×10^{-06}	2.62×10^{-11}
NAT2	7.89×10^{-03}	1.86×10^{-16}
HNF6	9.65×10^{-03}	4.04×10^{-02}
PCDHGA11	7.69×10^{-02}	0.926676
RAB11FIP1	4.69×10^{-01}	5.70×10^{-06}
SPAG11A	3.67×10^{-01}	5.53×10^{-01}
SIRPD	3.25×10^{-02}	4.27×10^{-01}
TOP1P2	2.12×10^{-01}	6.52×10^{-01}
WDR5	1.09×10^{-02}	4.33×10^{-10}
ZNF366	1.23×10^{-01}	5.03×10^{-01}
ZNF703	1.23×10^{-02}	1.22×10^{-17}
ZNRF3	1.40×10^{-09}	5.21×10^{-29}

Table S5. Mann-Whitney (MW) *p*-values correlating metastasis-enhancers expression with MSS *vs.* MSI CRC and Tumor (T) *vs.* Normal (N) using a cohort composed of 1436 cases.

Gene	MSS v. MSI-MW.pv	T v. N-MW.pv
MPDZ	4.47×10^{-01}	0.034502
DUSP14	8.90×10^{-04}	1.02×10^{-35}
SCEL	4.66×10^{-01}	3.82×10^{-08}
ANXA2P2	1.39×10^{-10}	3.41×10^{-03}
GLIS3	4.23×10^{-01}	1.39×10^{-01}
DOK5	0.875129	0.823477
VLDLR	1.04×10^{-02}	5.44×10^{-18}
CDC42BPA	0.260157	9.47×10^{-08}
USP32	7.89×10^{-04}	9.42×10^{-01}
PITPNC1	1.36×10^{-07}	2.06×10^{-06}
SEMG1	8.73×10^{-09}	4.94×10^{-01}
SMU1	1.01×10^{-11}	1.95×10^{-12}
ING1	2.09×10^{-02}	5.99×10^{-05}

Table S6. Mann-Whitney (MW) *p*-values correlating metastasis- enhancers expression with CRC grade using a cohort composed of 1436 cases. *p*-Values highlighted in bold indicate significant correlation ($p > 0.05$).

Gene	Grade 1 *vs.* Grade 2. MW.pv	Grade 1 *vs.* Grade 3. MW.pv	Grade 2 *vs.* Grade 3. MW.pv
MPDZ	**0.019342**	0.29412	0.097428
DUSP14	0.605543	0.062085	**0.007799**
SCEL	**0.039522**	0.333825	0.225055
ANXA2P2	0.322648	0.344263	**0.015006**
GLIS3	0.068606	**0.017196**	0.291099
DOK5	**0.000225**	**0.036341**	0.163315
VLDLR	0.824203	0.608901	0.675231
CDC42BPA	0.480159	0.385258	0.564302
USP32	0.420768	0.20917	0.383217
PITPNC1	0.35331	0.129961	0.237943
SEMG1	0.258852	0.566197	0.680923
SMU1	0.154207	0.98763	0.068939
ING1	0.311019	0.580271	0.644258

Table S7. Mann-Whitney (MW) *p*-values correlating metastasis-enhancers expression with CRC stage using a cohort composed of 1436 cases. *p*-Values highlighted in bold indicate significant correlation ($p > 0.05$).

Gene	St. I *vs.* St. II	St. I *vs.* St. III	St. I *vs.* St. IV	St. II *vs.* St. III	St. II *vs.* St. IV	St. III *vs.* St. IV
MPDZ	**0.045841**	**0.009583**	**0.001658**	0.477648	**0.038911**	0.182032
DUSP14	**0.002751**	**0.011843**	**0.017458**	0.556179	0.880672	0.752379
SCEL	**0.020656**	**0.005734**	**0.04278**	0.206618	0.860441	0.193857
ANXA2P2	**0.035557**	0.058363	0.077578	0.997691	0.872143	0.886247
GLIS3	**0.045841**	**0.000171**	0.101671	**0.038591**	0.615848	**0.012062**
DOK5	0.07008	**0.006356**	0.057107	0.130821	0.803482	0.393965
VLDLR	**0.029086**	0.051751	**0.005432**	0.750194	0.171961	0.186565
CDC42BPA	0.277474	0.169559	0.124249	0.30737	0.178417	0.667804
USP32	0.579347	0.244156	0.164499	0.521766	0.303395	0.685001
PITPNC1	0.073659	0.157487	0.156026	0.482447	0.723091	0.889343
SEMG1	0.953987	0.185351	0.182477	0.080427	0.055664	0.66923
SMU1	0.754104	0.997548	0.245083	0.732698	0.28963	0.142163
ING1	**0.021112**	**0.007786**	**0.006191**	0.377871	0.093281	0.449392

Table S8. Spearman's Rho correlation and *p* value for epithelial to mesenchymal transition and expression of 29 potential metastasis suppressor genes.

Gene	Spear. EMT. Corr. Rho	Spear. EMT. Corr. pv
ADRA1A	−0.11554	1.14×10^{-05}
ADRA1D	−0.10702	4.83×10^{-05}
ADRB3	−0.12734	1.29×10^{-06}
APOBEC3D	−0.04603	8.12×10^{-02}
BRF2	0.043368	1.00×10^{-01}
C20orf202	−0.08187	1.90×10^{-03}
TEX43	−0.12857	1.02×10^{-06}
CABIN1	0.006294	8.12×10^{-01}
CACNA1I	−0.18416	2.02×10^{-12}
CSMD1	−0.1708	7.28×10^{-11}
DIO3	−0.12678	1.44×10^{-06}
EPHX2	−0.31109	1.36×10^{-33}
FAM83F	−0.38216	3.78×10^{-51}
GP1BB	−0.11784	7.56×10^{-06}
KIAA1656	−0.11734	8.27×10^{-06}
LOC339593	−0.11113	2.44×10^{-05}
MCM8	−0.12744	1.27×10^{-06}
NAT1	−0.22961	1.24×10^{-18}
NAT2	−0.36097	1.96×10^{-45}
HNF6	−0.05085	5.40×10^{-02}
PCDHGA11	−0.15348	5.05×10^{-09}
RAB11FIP1	−0.30085	2.00×10^{-31}
SPAG11A	−0.0079	7.65×10^{-01}
SIRPD	0.055333	3.60×10^{-02}
TOP1P2	−0.04795	6.93×10^{-02}
WDR5	−0.30379	4.86×10^{-32}
ZNF366	−0.06771	1.03×10^{-02}
ZNF703	−0.32778	2.58×10^{-37}
ZNRF3	−0.24291	9.91×10^{-21}

Table S9. Spearman's Rho correlation and *p*-value for epithelial to mesenchymal transition and expression of 13 potential metastasis-enhancer genes.

Gene	Spear. EMT. Corr. Rho	Spear. EMT. Corr. pv
MPDZ	0.683395	3.43×10^{-198}
DUSP14	0.316919	7.25×10^{-35}
SCEL	0.062208	1.84×10^{-02}
ANXA2P2	0.016965	5.21×10^{-01}
GLIS3	0.308117	5.90×10^{-33}
DOK5	0.656223	1.33×10^{-177}
VLDLR	0.107559	4.42×10^{-05}
CDC42BPA	0.006847	0.795453
USP32	0.215219	1.65×10^{-16}
PITPNC1	0.000786	9.76×10^{-01}
SEMG1	−0.07814	3.05×10^{-03}
SMU1	−0.02528	0.338419
ING1	−0.04653	7.80×10^{-02}

Table S10. Metastatic CRC deleted genes' ontology and supporting evidence of their CRC involvement in cancer progression and metastasis. NA stands for none available.

Gene	Biological Process	References
ADRA1A	Growth inhibition	[18,47–51]
ADRA1D	Cell proliferation	[50–53]
ADRB3	Metabolic regulation	[53,54]
APOBEC3D	Viral immune response; retrotransposition inhibition	[20,55,56]
BRF2	Cell proliferation; hemopoiesis	[57,58]
C20orf202	Unknown	NA
TEX43	Unknown	NA
CABIN1	Inflammation and apoptosis regulation	[16,59]
CACNA1I	Calcium signaling; neuronal excitability	[60]
CSMD1	Tumor suppressor gene; unknown function	[14,61]
DIO3	Thyroid hormone inactivation	[25,62]
EPHX2	Lipid metabolism	[,15,63]
FAM83F	Suppressed in cancer; unknown function	[15,64]
GP1BB	Blood coagulation; cell adhesion	[23]
KIAA1656	Non-coding RNA; unknown function	NA
LOC339593	Non-coding RNA; unknown function	NA
MCM8	cellular response to DNA damage stimulus; DNA replication	[21,65]
NAT1	Biological oxidation; drug metabolism; chemical carcinogenesis	[66–68]
NAT2	Biological oxidation; drug metabolism; chemical carcinogenesis	[66,69]
HNF6	Cell differentiation; cell fate commitment, cell migration; glucose metabolism	[27,70–72]
PCDHGA11	Cell adhesion	[24]
RAB11FIP1	Negative regulation of adiponectin secretion; protein transport; endocytosis	[73,74]
SPAG11A	Unknown	NA
SIRPD	Unknown	NA
TOP1P2	Unknown	NA
WDR5	Chromatin organization; histone H3 acetylation; histone H3-K4 methylation	[22,75–76]
ZNF366	Negative regulation of estrogen receptor signaling pathway	[77,78]
ZNF703	Cell proliferation; cell migration; EMT regulation	[79,80]
ZNRF3	Negative regulation of WNT signaling pathway	[26]

Table S11. Metastatic CRC amplified genes' ontology and supporting. The evidence of their CRC involvement in cancer progression and metastasis.

Gene	Biological Process	References
ANXA2P2	Expressed pseudogene of unknown function	[81]
CDC42BPA	Cytoskeleton organization; cell migration	[82]
DOK5	MAPK cascade; neurite growth; tyrosine kinase signaling pathway	[83,84]
DUSP14	Inactivation of MAPK activity, peptidyl tyrosine dephosphorylation	[85,86]
SCEL	Embryo development	[87]
GLIS3	Transcriptional regulation from RNA polymerase II promoter	[88]
MPDZ	Cell adhesion; myelination	[89–91]
VLDLR	Cholesterol metabolism, cellular response to hypoxia and glucose starvation	[32]
USP32	Ubiquitin-dependent protein catabolism; protein de-ubiquitination	[33]
PITPNC1	Phospholipid transport; pro-angiogenic; signal transduction	[34,92]
SEMG1	Insemination; negative regulation of calcium ion import	[93]
SMU1	Genome integrity, regulation of DNA synthesis	[30]
ING1	Cell cycle; chromatin modification; negative regulation of cell growth	[31,94]
